# Divergent patterns of cognitive deficits and structural brain alterations between older adults in mixed-sex and same-sex relationships

**DOI:** 10.3389/fnhum.2022.909868

**Published:** 2022-09-02

**Authors:** Riccardo Manca, Anthony N. Correro, Kathryn Gauthreaux, Jason D. Flatt

**Affiliations:** ^1^Department of Life Sciences, Brunel University London, Uxbridge, United Kingdom; ^2^Mental Health Service, VA Ann Arbor Healthcare System, Ann Arbor, MI, United States; ^3^Department of Psychiatry, University of Michigan Health, Ann Arbor, MI, United States; ^4^National Alzheimer's Coordinating Center, Department of Epidemiology, University of Washington, Seattle, WA, United States; ^5^Department of Social and Behavioral Health, School of Public Health, University of Nevada, Las Vegas, Las Vegas, NV, United States

**Keywords:** cognitive decline, parahippocampal gyrus, same-sex relationship, sexual minority, National Alzheimer's Coordinating Center

## Abstract

**Background:**

Sexual minority (SM) older adults experience mental health disparities. Psychiatric disorders and neuropsychiatric symptoms (NPS) are risk factors for cognitive decline. Although older people in same-sex (SSR) compared to mixed-sex relationships (MSR) perform more poorly on cognitive screening tests, prior studies found no differences in rates of dementia diagnosis or neuropsychological profiles. We sought to explore the role of NPS on neurocognitive outcomes for SM populations. We compared cognitive performance and structural brain parameters of older adults in SSR and MSR.

**Methods:**

Data were originally collected at Alzheimer's Disease Research Centers (ADRCs). Inclusion criteria were: age of 55+ years, a study partner identified as a spouse/partner, and availability of T1-MRI brain volumes/thickness. Participants were labeled as either SSR or MSR based on their/their co-participant's reported sex. We identified 1,073 participants (1,037 MSR−555 cognitively unimpaired [CU]; 36 SSR−23 CU) with structural MRI data, Mini-Mental State Exam (MMSE), and Neuropsychiatric Inventory Questionnaire (NPI-Q) scores. A subset of the overall sample completed comprehensive neuropsychological assessment (*n* = 939; 908 MSR−494 CU; 31 SSR−22 CU). Covariates included in statistical models were age, sex, education, total intracranial volume, and apolipoprotein E genotype.

**Results:**

Multivariate general linear models showed significant diagnosis-by-relationship interaction effects on the left parahippocampal gyrus volume. After stratification by relationship group, only cognitively impaired (CI) MSR had significantly smaller left parahippocampal volumes than MSR-CU. The SSR group showed better episodic memory performance. Severity of neuropsychiatric symptoms was negatively associated with volume/thickness of bilateral fronto-temporal areas and with MMSE scores, predominantly in the MSR group.

**Conclusion:**

In our study, MSR participants presented with a more compromised cognitive profile than SSR participants. MSR-CI participants showed significantly smaller left medio-temporal volumes, a neural signature of AD. Neuropsychiatric symptoms predicted smaller fronto-temporal volumes in the MSR more consistently than in the SSR group. These findings may be due to unexplored protective factors against cognitive decline in SM elders. Indeed, social support has been proposed as a protective factor warranting future investigation.

## Introduction

Health disparities refer to dissimilar health outcomes, such as disease incidence, prevalence, or burden, across social or cultural groups (Carter-Pokras and Baquet, 2002). Lesbian, gay, bisexual, transgender, and queer or gender diverse (LGBTQ+) adults experience health disparities relative to cisgender (i.e., not transgender) and/or heterosexual adults (Caceres et al., 2017; Nelson and Andel, 2020). For example, a recent, large-scale (*n* = 1,659), electronic medical record review within a public mental health system in New York state indicated gay/bisexual men (relative to heterosexual men) had increased risk for diabetes, cardiometabolic diseases, depression, and anxiety, whereas lesbian/bisexual women (relative to heterosexual women) had increased risk for liver disease, substance misuse, hearing/vision impairment, and bipolar disorder (Rowan et al., 2022). Examination of United States (U.S.) public health datasets revealed increased prevalence of depressive disorders for lesbian and gay older adults; elevated alcohol misuse, tobacco use, and chronic health problems among lesbian and bisexual women in late midlife (50–64 years of age); and more prevalent obesity among older gay men (Dai and Meyer, 2019). One systematic review of 199 studies indicated sexual minority groups (LGB+) have greater risk for suicidal behaviors, pathological substance use, mood disorders, anxiety disorders, and other mental health conditions, relative to heterosexual groups (Plöderl and Tremblay, 2015). Generally, bisexual groups had greater symptomatology relative to lesbian and gay groups, revealing diverging risk/resilience pathways for unique sexual minority identities. Reported effect sizes were medium but ranged from small (alcohol/drug misuse) to large (suicidal ideation/behavior), with men generally showing larger effects.

Identity-related stressors are unique experiences of stigma, discrimination, and prejudice based on a person's actual or perceived identity, and exposure to those stressors is thought to underlie, or at least impact, the health disparities experienced by marginalized groups (Meyer, 2003; Hatzenbuehler, 2009). Lifetime discrimination and internalized heterosexism, or the turning inward of society's negative views toward non-heterosexual experiences, are strongly associated with mood disorders and psychological distress among LGBTQ+ adults and older adults (Newcomb and Mustanski, 2010; Fredriksen-Goldsen et al., 2013a,b; Hoy-Ellis and Fredriksen-Goldsen, 2017). Some possible mechanisms through which identity-related stressors could negatively affect physical health include allostatic load (i.e., changes to bodily systems and functions that result from protracted stress responses, such as chronic inflammation and abnormal hypothalamic-pituitary-adrenal (HPA) axis functioning) and neurotoxic effects of excessive endogenous stress hormones (McEwen and Stellar, 1993; Lupien et al., 2018). Regardless of one's actual or perceived identity/identities, cardiovascular diseases, metabolic conditions (such as diabetes), obesity, tobacco and alcohol use, and depression are risk factors for pathological cognitive aging (Alzheimer's Association, 2021). Taken together, LGBTQ+ adults appear to be at risk for accelerated cognitive decline (Correro and Nielson, 2019).

Few studies to date have explored the neurocognitive functioning of LGBTQ+ people in late-life, and the evidence for cognitive health disparities has been equivocal depending on outcome variables selected. Two U.S. population-wide studies showed higher rates of subjective cognitive complaints in LGBTQ+ older adults (Flatt et al., 2021; Fredriksen-Goldsen et al., 2021), which may be partially explained by mental health problems, such as depression (Flatt et al., 2018). Studies using cognitive screening tests suggest LGBTQ+ older adults experience greater cognitive impairment than older heterosexual people (Hsieh et al., 2021; Liu et al., 2021). Yet, our prior studies with a nationwide clinical dataset found no differences in diagnosis rates of mild cognitive impairment (MCI) and all-cause dementia between people in same- (SSR; “lesbian/gay”) vs. mixed-sex (MSR; “heterosexual”) relationships (Perales-Puchalt et al., 2019). Moreover, both MSR and SSR groups showed similar functional, clinical, and cognitive profiles at baseline, and their annual rates of change in cognition were not significantly different, except for a possible incidental finding in which people in MSR who had dementia at baseline declined more quickly on measures of attention/working memory relative to people in SSR with dementia at baseline (Correro et al., 2021). Finally, another study using a U.S. clinical dataset examined both neuropsychological functioning, neuropsychiatric symptoms, and brain volume (T1-weighted magnetic resonance imaging [MRI]) in people in SSR vs. those in MSR, with and without Alzheimer's disease (AD; Manca and Venneri, 2020). Although the MSR and SSR groups had similar neuropsychological profiles, the SSR group presented with more severe neuropsychiatric symptoms. Divergent patterns of gray matter atrophy were also found, possibly suggesting unique brain aging parameters for sexual minority adults. Individuals in SSR had more pronounced atrophy in prefrontal (PFC) and posterior cingulate (PCC) regions (areas associated with the default mode network), whereas participants in MSR had atrophic bilateral medial temporal lobes (MTL) and right insula/superior temporal gyrus (STG; areas essential for learning and memory). For the SSR group, neuropsychiatric symptoms were negatively associated with volumes of bilateral medial PFCs and of the left insula/STG. Those regions have been previously implicated in AD with behavioral disturbance (i.e., comorbid neuropsychiatric symptoms; Wang X. et al., 2019; Boublay et al., 2020). However, no data were available to ascertain whether such subtle differences in behavioral and neural alterations could represent consequences of minority stress.

Recently, Nicholson et al. (2022) incorporated minority stress literature (e.g., Meyer, 2003; Hatzenbuehler, 2009; Pachankis, 2015; Feinstein, 2020) and neural transdiagnostic models of posttraumatic stress disorder, depression, and anxiety (e.g., Menon, 2011; Nicholson et al., 2020) to devise viewpoints on the neural correlates of sexual minority stress. They reviewed 12 studies of functional, structural, and metabolic neural foundations of sexual orientation. They highlighted alterations to key nodes in default mode (ventromedial PFC, PCC/precuneus), salience (insula, dorsal anterior cingulate cortex, amygdala, brainstem, periaqueductal gray), and central executive (dorsolateral PFC, posterior parietal cortex, cerebellum) networks, that were suggested to be due to sexual minority stress. They proposed a minority mosaic framework in which a person (i.e., an individual tile in a mosaic) can be considered within their sociocultural contexts (i.e., the mosaic as a whole) and suggested that future neuroimaging research should implement multivariate machine learning approaches and graph theoretical network-based analyses to better understand sexual minority stress and its effects on neuropsychiatric functioning. In light of Nicholson et al. (2022) models, we sought to strategically re-examine the nationwide AD and related dementia dataset we previously used.

The primary objectives of this study were (1) to replicate prior findings (i.e., differential patterns of neuropsychiatric symptoms and brain structure alterations between participants in SSR and MSR); and (2) to evaluate whether neuropsychiatric symptoms predict cognitive and structural outcome measures (Manca and Venneri, 2020; Nicholson et al., 2022). An ancillary goal was to explore possible differences in outcome measures (screening vs. comprehensive assessments). We hypothesized that those in SSR would have worse mental status exams relative to MSR (Hsieh et al., 2021; Liu et al., 2021), but we expected to find no statistically significant differences between these groups on comprehensive neuropsychological testing (Correro et al., 2021). We anticipated divergent structural patterns (PFC and PCC involvement in SSR; MTL and right insula/STG involvement in MSR). We expected the SSR group to report more neuropsychiatric symptoms and that their symptoms would be differentially associated with volumes in fronto-temporal areas—those associated with the salience network (cf. Manca and Venneri, 2020; Nicholson et al., 2022).

## Materials and methods

### Participants

Participants' data were obtained from the National Alzheimer's Coordinating Center (NACC) Uniform Data Set (UDS), which is a standardized clinical dataset originally collected across the U.S. at Alzheimer's Disease Research Centers (ADRCs) funded by the National Institute of Aging (Beekly et al., 2007; Besser et al., 2018). All contributing ADRCs are required to obtain informed consent from their participants and to maintain their own separate Institutional Review Board (IRB) reviews/approvals from their institutions prior to submitting data to NACC. Data used herein were collected between September 2005 and March 2021. The following inclusion criteria were used to select participants for this study: (1) availability of data on regional volume and thickness derived from the pre-processing of T1-weighted MRI scans acquired within 1 year from the closest UDS visit; (2) availability of a study partner identified as a spouse, partner, or companion; (3) 55 years of age or older. Following procedures used in previous studies (e.g., Perales-Puchalt et al., 2019; Manca and Venneri, 2020), we assigned participants to the SSR group if they had at least one visit where their co-participant was their spouse, partner, or companion and the co-participant reported having the same sex as the participant, whereas participants reporting a different sex than their co-participant were labeled as MSR. This strategy is commonly used to identify non-heterosexual participants in large databases not designed for this purpose (Umberson et al., 2015; Perales-Puchalt et al., 2019; Manca and Venneri, 2020; Liu et al., 2021).

The first author screened a total of 1,880 entries for 1,162 participants in which pre-processed MRI volumetric/thickness data were available. Among these, 1,119 participants were identified with MRI assessment available within 1 year from the closest UDS visit. We excluded 32 participants who were younger than 55 years, 11 without cognitive screening data, and three due to lack of data on education. The final sample included 1,073 participants assessed between September 2005 and October 2018: 1,037 in the MSR group and 36 in the SSR group.

### Clinical and cognitive data

Participants' demographic characteristics, cognitive status, and mental health conditions were extracted from NACC UDS databases. The global score of the CDR^®^ Dementia Staging Instrument (Morris, 1993), a tool used across ADRCs to assess severity of cognitive decline, was included in this study to label participants as cognitively impaired (CDR^®^ ≥ 0.5) or unimpaired (CDR^®^ < 0.5). The MSR group comprised 555 cognitively unimpaired and 482 impaired participants, while the SSR group included 23 cognitively unimpaired and 13 impaired participants.

Depending on availability across versions of the UDS (Morris et al., 2006; Weintraub et al., 2009, 2018), either Mini-Mental State Examination (MMSE) or Montreal Cognitive Assessment (MoCA) scores were extracted. MoCA scores were converted into MMSE scores following published norms (Roalf et al., 2013) to enable group-wise analyses. A range of neuropsychological tests was also available, with variable rates of missing data across tests, for a subset of the participant sample (*n* = 939). Tests from the UDS version 2 were: Logical Memory—immediate and delayed recall (for learning/memory), Digit Span Forward and Backward for attention/working memory, Category Fluency (average of total correct items across two categories: animals and vegetables) for semantic fluency, and Trail Making Test Parts A and B for processing speed/mental flexibility. For participants assessed with the UDS version 3, learning/memory was evaluated with the Craft Story 21 (immediate and delayed recall) and working memory with Number Span (forward and backward). We applied validated conversion tables (Monsell et al., 2016) to equate those scores with Logical Memory and Digit Span scores, respectively. Additionally, we used the total scores from the Neuropsychiatric Inventory Questionnaire (NPI-Q) to measure the severity of neuropsychiatric symptoms (Kaufer et al., 2000).

Data on apolipoprotein E (APOE) genotype were also extracted. Genetic data were analyzed by either ADRCs, the Alzheimer's Disease Genetics Consortium (ADGC), or National Institute of Aging Genetics of Alzheimer's Disease Data Storage Site (NCRAD) and subsequently shared with NACC.

### MRI data

The NACC imaging database includes a convenience sample of MRI data. Those data were acquired with different protocols and parameters across centers. Volume (in cubic centimeters) and cortical thickness (in millimeters) for 32 bilateral gray matter (GM) regions (31 for cortical thickness since only the volume was calculated for the hippocampus, see Supplementary Table 1) were provided to NACC by the IDeA Lab at the University of California, Davis. T1-weighted structural MRI scans were acquired at multiple centers using 3.0 and 1.5 Tesla scanners (GE, Siemens, and Phillips). Structural scans were processed based on the Advanced Normalization Tools (ANTs) toolkit and thickness pipeline (Das et al., 2009). All total volumes [i.e., GM, white matter, cerebrospinal fluid, and total intracranial volume (TIV)] were calculated using the ADNI four-tissue segmentation protocol (http://adni.loni.usc.edu/methods/mri-tool/), and hippocampal volume was calculated using the EADC-ADNI harmonized protocol (Frisoni et al., 2015).

### Statistical analyses

All statistical analyses on clinical variables were carried out in SPSS version 26 (IBM, Chicago, IL, USA). Demographic and global brain tissue volumes of SSR and MSR groups were compared using Mann-Whitney *U* tests. To compare cognitive performance and brain structural parameters between SSR and MSR participants (i.e., our primary aim), a set of multivariate general linear models were used to investigate the effects (main and interaction) of clinical diagnosis and relationship type (SSR vs. MSR) on regional GM volume, cortical thickness, and cognitive performance (where available). Covariates with potential confounding effects on either cognition or brain health were included in the models: age, sex, education, total intracranial volume, and APOE genotype (ε4 carriers vs. non-carriers). Additionally, exploratory analyses were computed with (1) interactions between relationship type and all covariates, and (2) pair-wise interactions between all covariates included in the models. Separate multivariate general linear models adjusting for the same covariates were used as *post-hoc* analyses to compare cognitive and brain structural outcome measures between SSR and MSR groups stratified by diagnosis and between clinical diagnosis stratified by relationship type.

Additionally, a set of analyses were carried out to investigate the secondary aim of this study (i.e., quantify potential impact of minority stress, viz. NPI-Q scores, on neurocognitive outcome measures across relationship groups). First, NPI-Q total scores were compared between MSR and SSR groups using the Mann-Whitney *U* test. Differences in rates of individual neuropsychiatric symptoms between relationship groups were assessed using the chi-square test and the Fisher's exact test. These analyses were repeated after stratifying the sample by diagnosis. Finally, multivariate general linear models adjusting for the same covariates as those reported above were also used to investigate the association between NPI-Q total scores and cognitive and structural brain measures (i.e., volume and cortical thickness) in the whole sample and in the two relationship groups independently. We applied the Bonferroni correction to account for multiple comparisons. Figures were created using ggplot2 (Wickham, 2016) and ggseg (Mowinckel and Vidal-Piñeiro, 2020) packages for R (www.r-project.org/).

## Results

### Descriptive statistics

The MSR and SSR groups were not significantly different in terms of demographic, clinical, and global brain structural characteristics (Table 1). Similar results were obtained when we compared characteristics of SSR and MSR individuals with available neuropsychological data (Supplementary Table 2).

**Table 1:** Demographic, clinical, and neural characteristics of the sample.

**Variable**	**SSR (*n* = 36)**	**MSR (*n* = 1,037)**	***U***	***p***
Age	78.00 (16)	76.00 (14)	0.96	0.336
Education	18.00 (4)	16.00 (4)	1.30	0.195
Sex (F/M)	21/15^a^	458/579^a^	2.83^b^	0.093
ApoE (ε4+/ε4–)	14/22^a^	447/590^a^	0.25^b^	0.615
Diagnosis (CU/CI)	23/13^a^	555/482^a^	1.51^b^	0.220
UDS version (1/2/3)	2/23/11^a^	120/691/226^a^	2.36^b^	0.308
CDR	0.00 (0.5)	0.00 (0.5)	−1.80	0.072
GMV (cm^3^)	502.07 (67.74)	497.69 (75.87)	0.25	0.803
WMV (cm^3^)	410.49 (78.73)	410.84 (80.21)	−0.53	0.594
CSFV (cm^3^)	283.13 (79.49)	296.96 (74.15)	−1.10	0.273
TIV (cm^3^)	1201.47 (185.10)	1212.09 (182.88)	−0.85	0.396

Values are medians (interquartile range) analyzed with Mann-Whitney *U* test, unless otherwise specified.

CDR, Clinical Dementia Rating Scale; CI, cognitively impaired; CSFV, cerebrospinal fluid volume; CU, cognitively unimpaired; GMV, gray matter volume; TIV, total intracranial volume; WMV, white matter volume; UDS: Uniform Data Set.

^a^Frequency.

^b^χ^2^.

### Brain structure

**Figure 1:**
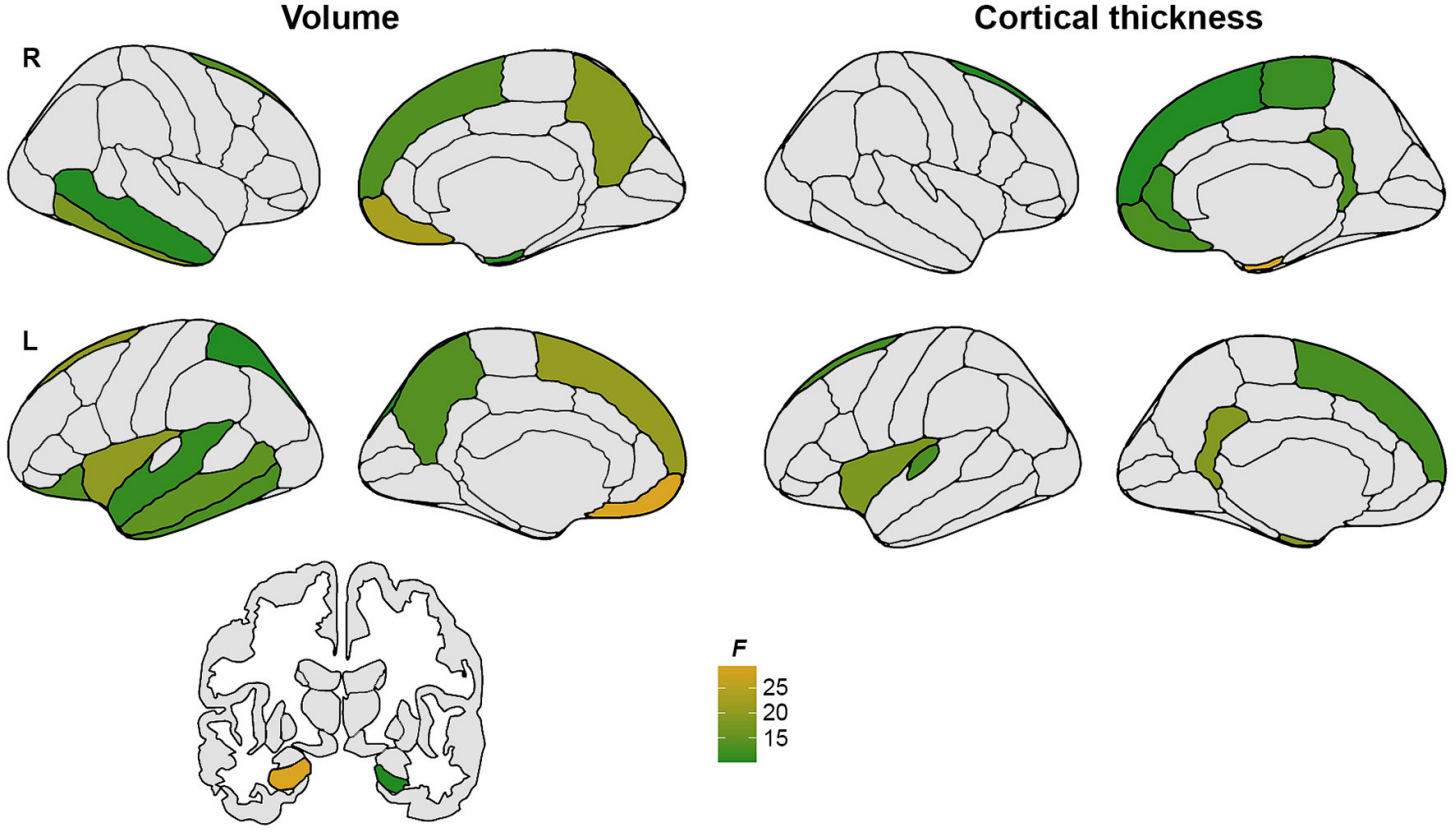
Gray matter regions showing significantly lower volume and thickness values in CI compared to CU participants.

Generally, unimpaired participants presented with larger GM volumes and higher cortical thickness values (Figure 1) and with no significant brain structural differences between SSR and MSR groups.

Significant diagnosis-by-relationship interaction effects were observed for the volume of the left parahippocampal gyrus (Supplementary Table 3). Significant *post-hoc* comparisons showed that cognitively unimpaired MSR had larger left parahippocampal gyri than cognitively unimpaired SSR [*F*(6, 540) = 6.45, *p* = 0.011], while the SSR-CI group had a larger volume than the MSR-CI group [*F*(6, 457) = 4.26, *p* = 0.039]. Also, left parahippocampal gyrus volumes were significantly different between the MSR-CI group compared to MSR-CU group [*F*(6, 999) = 69.92, *p* < 0.001], but there was no difference in that region for the SSR groups.

A significant interaction effect emerged between relationship type and TIV for cortical thickness of the left middle temporal gyrus [*F*(13, 1,059) = 10.27, *p* = 0.001]. In particular, while no association was detected between TIV and cortical thickness in the MSR group (ρ = −0.040, *p* = 0.196), a significant negative association (ρ = −0.396, *p* = 0.017) was detected in the SSR group (Supplementary Figure 1). Significant interactions were also found between education and TIV, for the volume of the left isthmus cingulate cortex [*F*(18, 1,054) = 10.15, *p* = 0.001; Supplementary Figure 2] and for thickness of the left pars triangularis of the inferior frontal gyrus [*F*(18, 1,054) = 11.05, *p* < 0.001; Supplementary Figure 3]. In general, regional brain volumes and cortical thickness values were equivalent between relationship groups and between male and female participants (Supplementary Tables 4, 5).

### Cognitive performance

**Figure 2:**
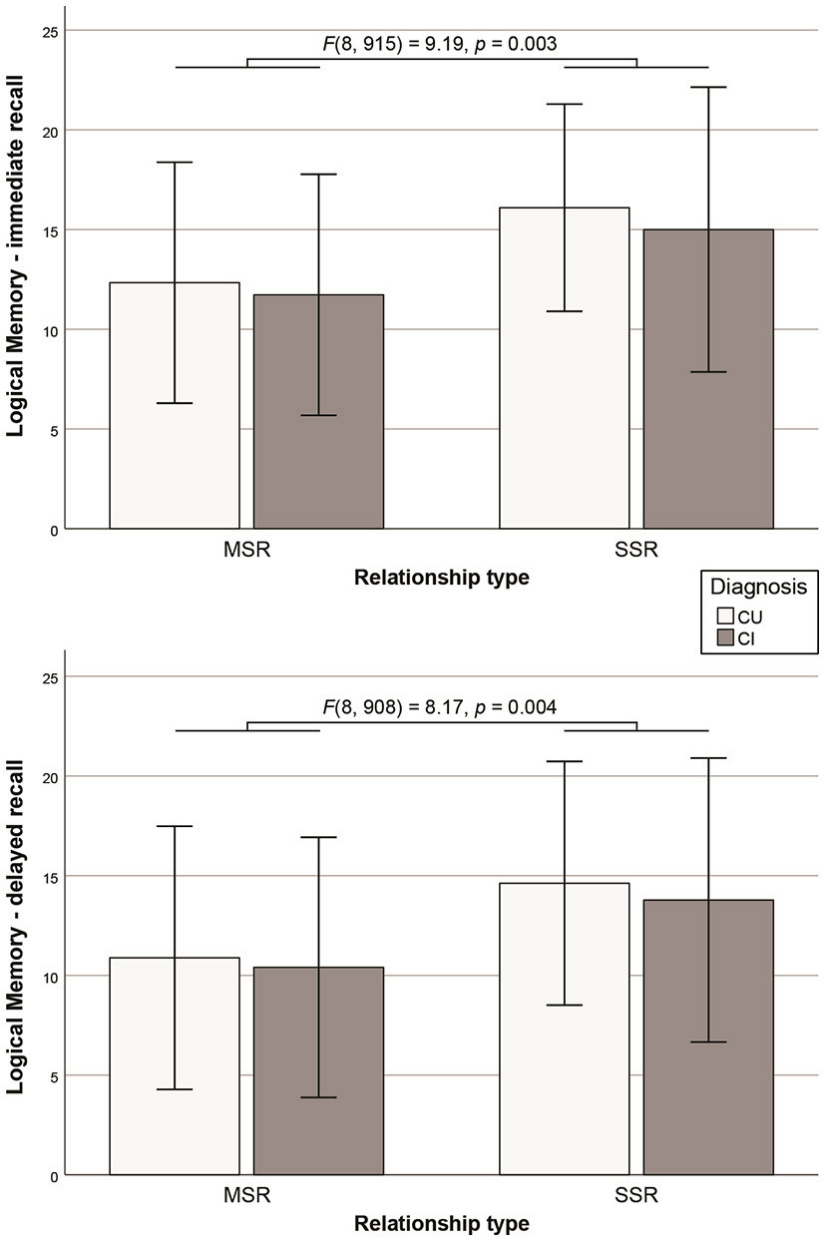
Significant difference in the Logical Memory Test immediate and delayed recall performance between SSR and MSR groups (error bars representing standard deviations).

On cognitive outcome measures, cognitively unimpaired participants scored higher than cognitively impaired participants on the MMSE [*F*(8, 930) = 30.76, *p* < 0.001]. The SSR group performed better than the MSR group on a test of verbal long-term memory (i.e., Logical Memory—immediate and delayed recall (Figure 2). All other comparisons and diagnosis-by-relationship interactions were not significant (Table 2).

**Table 2:** Diagnosis-by-relationship effects on cognitive performance (from general linear models including all covariates).

**Variable**		**SSR-CU**		**SSR-CI**		**MSR-CU**		**MSR-CI**	***F***	***p***
	***n***		***n***		***n***		***n***			
MMSE	22	29.5 (1)	9	26.0 (6)	494	29.0 (1)	414	26.0 (6)	1.34	0.248
LMI	21	18.0 (10)	9	16.0 (9)	490	12.0 (9)	404	12.0 (9)	0.01	0.933
LMD	21	17.0 (10)	9	15.0 (9)	487	11.0 (10)	400	11.0 (11)	0.00	1.000
DSF	22	7.0 (2)	9	7.0 (3)	492	6.0 (2)	408	6.5 (1)	0.60	0.438
DSB	22	5.5 (2)	9	5.0 (4)	492	4.0 (2)	408	4.0 (2)	0.75	0.386
SFA	22	20.0 (12)	9	20.0 (13)	493	17.0 (11)	411	16.0 (9)	0.41	0.522
SFV	22	15.5 (10)	9	12.0 (14)	493	12.0 (7)	406	12.0 (7)	0.83	0.361
TMTA	21	29.0 (19)	9	35.0 (18)	469	35.0 (26)	399	36.0 (27)	0.17	0.681
TMTB	20	66.5 (45)	8	99.5 (46)	433	88.0 (89)	351	100.0 (103)	0.03	0.856

Values are uncorrected medians (interquartile range), unless otherwise specified.

DSB, Digit Span Backward; DSF, Digit Span Forward; LMD: Logical Memory—Delayed Recall; LMI, Logical Memory—Immediate Recall; SFA, semantic fluency—animals; SFV, semantic fluency—vegetables; TMTA, Trail Making Test—Part A; TMTB, Trail Making Test—Part B.

For the cognitive outcome measures, no significant interaction effects were found either between relationship type and covariates or between any of the covariates included in the models. Cognitive test scores were, in general, similar between relationship groups and between male and female participants (Supplementary Table 6).

### Neuropsychiatric symptoms

No statistically significant differences between MSR and SSR groups were found in either NPI-Q total scores (*U* = −1.88, *p* = 0.059) or in rates of individual neuropsychiatric symptoms (Table 3). A similar pattern of results was observed when cognitively unimpaired and cognitively impaired groups were analyzed separately (Supplementary Table 7), with severity of neuropsychiatric symptoms equivalent between relationship groups: cognitively unimpaired MSR vs. SSR (*U* = 0.07, *p* = 0.943) and cognitively impaired MSR vs. SSR (*U* = −1.81, *p* = 0.071).

**Table 3:** Differences in behavioral profiles between MSR and SSR groups.

**Variable**	**SSR (*n* = 36)**	**MSR (*n* = 1,005)**	**χ^2^**	***p***
NPI-Q total score	0.00 (1)	0.00 (3)	−1.88^a^	0.059
Delusions (Y/N)	1/35 (2.8%)	48/957 (4.8%)	–^b^	1.000
Hallucinations (Y/N)	0/35(0.0%)^c^	29/976 (2.9%)	–^b^	0.620
Agitation (Y/N)	4/32 (11.1%)	147/858 (14.6%)	0.34	0.556
Depression (Y/N)	3/33 (8.3%)	197/808 (19.6%)	2.84	0.092
Anxiety (Y/N)	5/31 (13.9%)	218/787 (21.7%)	1.26	0.262
Euphoria (Y/N)	1/35 (2.8%)	19/986 (1.9%)	–^b^	0.509
Apathy (Y/N)	2/34 (5.6%)	175/830 (17.4%)	3.46	0.063
Disinhibition (Y/N)	1/35 (2.8%)	95/910 (9.5%)	–^b^	0.244
Irritability (Y/N)	5/31 (13.9%)	229/776 (22.8%)	1.58	0.209
Motor disturbance (Y/N)	1/35 (2.8%)	66/939 (6.6%)	–^b^	0.724
Night-time behaviors (Y/N)	5/31 (13.9%)	169/835 (16.8%)^d^	0.22	0.642
Appetite disturbance (Y/N)	3/33 (8.3%)	114/890 (11.4%)^d^	–^b^	0.789

For the NPI-Q total score, values are medians (interquartile ranges).

For all other variables, values are frequencies of neuropsychiatric symptoms (percentage).

^a^Mann-Whitney *U* test.

^b^Fisher's exact test.

^c^*n* = 35.

^d^*n* = 1,004.

### Relationships between brain structures, cognitive outcomes, and neuropsychiatric symptoms

The total NPI-Q score was negatively associated with GM volume in bilateral temporal, left lateral orbitofrontal, and right entorhinal cortices (Table 4). Similarly, higher NPI-Q total scores were negatively associated with thickness of right entorhinal and bilateral temporal and cingulate cortices. Those results were replicated in the MSR group after conducting separate analyses on the MSR and SSR groups (Table 4). NPI-Q total scores were also negatively associated with MMSE scores (both in the whole sample and in the MSR group alone). No significant associations between severity of neuropsychiatric symptoms and any other cognitive outcome measures were observed (Table 5).

**Table 4:** Significant associations between NPI-Q total scores and regional GM volume and cortical thickness values (Bonferroni-corrected significance *p* < 0.0016).

**Variable**	**Whole sample**			**SSR**			**MSR**		
	**b**	***p***	** ${\eta_{\text{p}}}^{2}$ **	**b**	***p***	** ${\eta_{\text{p}}}^{2}$ **	**b**	***p***	** ${\eta_{\text{p}}}^{2}$ **
**Volume**									
Left ITG	−0.06	<0.001	0.02	−0.26	<0.001	0.40	−0.06	<0.001	0.02
Right ITG	−0.06	<0.001	0.02	−0.19	0.007	0.27	−0.06	<0.001	0.02
Left MTG	−0.06	<0.001	0.02	−0.34	<0.001	0.47	−0.05	<0.001	0.01
Right MTG	−0.06	<0.001	0.02	−0.26	0.003	0.31	−0.06	<0.001	0.01
Right entorhinal cortex	−0.02	0.001	0.01	−0.05	0.210	0.06	−0.02	0.001	0.01
Left lateral OFC	−0.03	<0.001	0.01	−0.07	0.090	0.11	−0.02	0.001	0.01
**Cortical thickness**									
Left ITG	−0.02	<0.001	0.01	−0.07	0.014	0.23	−0.02	<0.001	0.01
Right ITG	−0.02	<0.001	0.01	−0.05	0.082	0.12	−0.02	<0.001	0.01
Left MTG	−0.01	<0.001	0.01	−0.05	0.054	0.15	−0.01	<0.001	0.01
Left STG	−0.01	<0.001	0.01	−0.05	0.002	0.32	−0.01	<0.001	0.01
Right STG	−0.01	<0.001	0.01	−0.04	0.014	0.23	−0.01	<0.001	0.01
Right entorhinal cortex	−0.02	<0.001	0.01	−0.05	0.136	0.09	−0.02	0.001	0.01
Left fusiform gyrus	−0.02	<0.001	0.01	−0.07	0.002	0.33	−0.01	0.002	0.01
Right fusiform gyrus	−0.02	<0.001	0.02	−0.06	0.010	0.25	−0.02	<0.001	0.01
Left PCC	−0.01	0.005	0.01	−0.05	0.001	0.36	−0.1	0.012	0.01
Right PCC	−0.01	<0.001	0.01	−0.03	0.095	0.11	−0.01	0.001	0.01
Left ICC	−0.02	<0.001	0.02	−0.03	0.042	0.16	−0.02	<0.001	0.02
Right ICC	−0.01	<0.001	0.01	−0.03	0.105	0.11	−0.01	<0.001	0.01

ICC, isthmus cingulate cortex; ITG, inferior temporal gyrus; MTG, middle temporal gyrus; OFC, orbitofrontal cortex; PCC, posterior cingulate cortex; STG, superior temporal gyrus.

**Table 5:** Associations between NPI-Q total scores and performance on cognitive tests (Bonferroni-corrected significance *p* < 0.006).

**Variable**	**Whole sample**				**SSR**				**MSR**			
	***n***	**b**	***p***	** ${\eta_{\text{p}}}^{2}$ **	***n***	**b**	***p***	** ${\eta_{\text{p}}}^{2}$ **	***n***	**b**	***p***	** ${\eta_{\text{p}}}^{2}$ **
MMSE	914	−0.27	<0.001	0.05	31	−0.46	0.061	0.17	883	−0.27	<0.001	0.05
LMI	900	0.05	0.474	<0.01	30	−1.00	0.157	0.11	870	0.06	0.390	<0.01
LMD	893	−0.01	0.851	<0.01	30	−1.25	0.106	0.14	863	−0.00	0.981	<0.01
DSF	907	0.02	0.243	<0.01	31	−0.16	0.338	0.05	876	0.02	0.207	<0.01
DSB	907	0.01	0.792	<0.01	31	−0.19	0.207	0.08	876	0.01	0.703	<0.01
SFA	910	0.12	0.150	<0.01	31	−0.09	0.916	<0.01	879	0.12	0.147	<0.01
SFV	905	−0.00	0.963	<0.01	31	−0.10	0.883	<0.01	874	−0.00	0.976	<0.01
TMTA	863	−0.20	0.538	<0.01	30	−1.59	0.593	0.02	833	−0.18	0.581	<0.01
TMTB	789	−1.17	0.215	<0.01	28	−1.01	0.804	<0.01	761	−1.18	0.220	<0.01

DSB, Digit Span Backward; DSF, Digit Span Forward; LMD, Logical Memory—Delayed Recall; LMI, Logical Memory—Immediate Recall; SFA, semantic fluency—animals; SFV, semantic fluency—vegetables; TMTA, Trail Making Test—Part A; TMTB, Trail Making Test—Part B.

## Discussion

A significant interaction emerged between diagnosis and relationship type (SSR/MSR) for left parahippocampal gyrus volumes, such that cognitively unimpaired participants in the SSR group had smaller left parahippocampal gyrus volumes relative to cognitively unimpaired MSR participants, while the opposite difference was found when comparing cognitively impaired participants. Yet, the SSR group had better verbal long-term memory performance than the MSR group overall. Severity of neuropsychiatric symptoms was associated with smaller volume/thickness values primarily in the MSR group and with lower MMSE scores in the MSR group only.

When the left parahippocampal gyrus volume was investigated in the SSR and MSR groups separately, no significant differences between cognitively impaired and unimpaired SSR participants were observed. This finding had already been highlighted by Manca and Venneri (2020), despite some methodological differences with that study (e.g., age, size and matching of samples, and time allowed between UDS and MRI scanning visits). By contrast, significantly smaller left parahippocampal volumes were found in the cognitively impaired MSR group. Hippocampal and parahippocampal regions are essential for learning and memory (Köhler et al., 1998) and the left medio-temporal lobe is a brain region particularly affected by AD (e.g., Berron et al., 2020). Although the cognitively impaired groups were not solely comprised of participants with AD, the NACC dataset and ADRCs broadly attempt to oversample people with AD or those who may develop AD. The cognitively impaired MSR group may better reflect that demographic since a larger, albeit not statistically significant, percentage of the MSR sample were cognitively impaired (46.4%) relative to the SSR group (36.1%). However, no major differences in cognitive performance could be detected when exploring interaction effects, in line with a previous study (Manca and Venneri, 2020), suggesting that the sizes of the cognitively impaired groups across relationship types do not contribute meaningfully to our interpretation of the results.

Given the association between episodic memory functioning and integrity of MTL areas, the better Logical Memory Test performance coupled with the smaller left parahippocampal gyri in cognitively unimpaired SSR participants suggests greater cognitive reserve (defined as preserved cognitive functions despite brain changes related to disease processes or normal aging; Stern et al., 2020) in this group. The cognitively impaired SSR group may show signs of resilience because their immediate memory scores approximated those of the cognitively unimpaired SSR and MSR groups. Coupled with their left parahippocampal GM volumes similar to, if not larger than, the cognitively unimpaired SSR and MSR groups, these findings suggest more preserved neurocognitive functioning, even in the presence of possible cognitive impairment, of adults in SSR. It must be noted that other recent studies support this interpretation. In a population-wide study of aging in Canada (baseline ages ranged from 45 to 85 years), non-heterosexual participants outperformed heterosexual participants on a test of episodic memory (Stinchcombe and Hammond, 2021). Baseline cognitive functioning and trajectories of cognitive decline were not different in another study that compared SSR and MSR participants using the NACC (Correro et al., 2021). Ultimately then, people in SSR may be resilient to the hypothetical, negative effects of minority stress on neurocognitive functioning. In fact, greater risk of cognitive impairment in people in SSR was only found by one study that used a single screening test (Liu et al., 2021), whereas no differences in rates of clinical diagnosis of dementia have been observed between people in SSR compared to those in MSR in NACC data (Perales-Puchalt et al., 2019).

Apart from differences in episodic memory performance, participants in either MSR or SSR appeared to have similar neurocognitive profiles. The impact of the covariates included in the statistical models was also similar across the two relationship groups, except for an unexpected negative association between TIV and the left middle temporal gyrus thickness in the SSR group only. Although the relevance of this association appears to be unclear, considering the very similar group-level TIV values in SSR and MSR groups, it may be argued that its impact on the significant findings of this study (i.e., relationship-by-diagnosis interaction effect on the left parahippocampal gyrus volume) is highly unlikely.

Since alterations in the processing and regulation of emotions are thought to mediate the impact of minority stress on the health of non-heterosexual people (Hatzenbuehler, 2009), we expected the SSR group to present with more severe neuropsychiatric symptoms (i.e., higher NPI-Q scores) and that these would be negatively associated with regional GM volume and cortical thickness in the sexual minority group (Manca and Venneri, 2020). However, contrary to our hypothesis, the two relationship groups had comparable behavioral profiles and rates of specific neuropsychiatric symptoms. The severity of neuropsychiatric symptoms was associated with reduced brain structural integrity in the whole sample, but this finding appeared to be driven mainly by the MSR group. In particular, the impact of behavioral alterations was isolated to brain regions, such as temporal, orbitofrontal, and cingulate areas, that are involved in emotional processing (Damasio et al., 2000; Kim and Hamann, 2007) and memory functions (Squire and Zola-Morgan, 1991; Maddock et al., 2001; Cheung and Chan, 2003). This finding appears to corroborate what emerged from previous investigations that neuropsychiatric symptoms are associated with reduced brain structural integrity primarily in prefrontal areas, such as orbitofrontal, dorsolateral, and anterior cingulate cortices (Bruen et al., 2008; Boublay et al., 2020; Manca and Venneri, 2020). When relationship groups were investigated separately, this pattern was evident in the MSR group, while higher NPI-Q scores were associated only with the volume of left lateral temporal areas in SSR participants. Similarly, severity of neuropsychiatric symptoms was associated with more compromised global cognition only in the whole sample and in the MSR group. These findings suggest that neurocognitive health may be more preserved in the SSR group, and the scarcity of associations between behavioral alterations and both neural and cognitive outcome measures in the SSR group may be related to effective coping. This speculation cannot be corroborated further by our data, considering that only NPI-Q was available as a variable considered to capture the potential effects of minority stress. Therefore, the lack of any data regarding minority stress and resilience more specifically prevents any definite conclusions on the role played by such variables.

Overall, our findings were inconsistent with our hypotheses of greater neurocognitive impairment in the SSR group, which would be expected as one of the possible consequences of minority stress in older adults (Hsieh et al., 2021; Liu et al., 2021). A possible speculative explanation of this finding may be related to resilience against cognitive decline in non-heterosexual older adults. Resilience factors in the minority stress model include group- and individual-level processes that may be identity-specific (e.g., LGBTQ+ community connectedness) or general (e.g., personal agency; Meyer, 2003). Similarly, dementia risk is moderated by interindividual factors, such as social connectedness. In a population-based cohort study, older adults' social network/support was protective against dementia related to cardiometabolic diseases (e.g., diabetes, stroke, heart disease; Wang Z. et al., 2019). A systematic review of longitudinal, population-based, observational dementia studies indicated social engagement (broadly construed) reduces risk for dementia diagnosis (Di Marco et al., 2014). The roles of marriage and living arrangement (alone or not alone) on dementia and minority stress have been more complex. Indeed, other work has suggested that marital status, as opposed to being in a romantic relationship, protects against cognitive decline (Sundström et al., 2014; Liu et al., 2021). Future studies are needed to discern whether sexual minority older adults without spouses, partners, or companions are protected from minority stress effects on neurocognition.

### Limitations

A first limitation of this study is the small sample size for the SSR groups, especially those with cognitive impairment, so our results may have been influenced by the healthy volunteer bias (Lindsted et al., 1996). We cannot rule out that the uneven sample sizes of the two relationship groups may have influenced differences in either cognitive or cerebral outcome measures. Figure 2 depicts error bars of similar sizes substantially overlapping across groups. Consistently, Supplementary Tables 4–6 show similar standard deviations for all cognitive and neural outcome measures between the relationship groups. Thus, some of the findings, such as better long-term memory performance in the SSR group, may be a consequence of the small sample size of this group and/or a selection bias (determined by data availability). In fact, the MSR group included a higher proportion of cognitively impaired participants than the SSR group, although this discrepancy was not statistically significant, and no significant differences were found for the total MMSE scores between two relationship groups. That said, a trend for more prevalent cognitive impairment in the MSR group might have contributed to poorer episodic memory performance.

Moreover, no data on participants' self-identified sexual orientation was available in the NACC database. This issue has been circumvented by selecting participants in either SSR or MSR, a strategy that has been extensively used to identify participants highly likely to be non-heterosexual in large databases designed with no such specific purpose (Umberson et al., 2015), including retrospective analyses of the NACC UDS (e.g., Perales-Puchalt et al., 2019; Manca and Venneri, 2020; Liu et al., 2021). By adopting this approach, it is not possible to rule out the misclassification of some participants, i.e., some participants in the SSR group may identify as heterosexual and some people in the MSR group may identify as non-heterosexual. These sub-samples, however, are estimated to be small and to have very little influence on the findings of our study. For example, Taylor and Gonzales (2022) used data from the 2013–2018 National Health Interview Survey, which is a health survey of the civilian, non-institutionalized, U.S. population, and they identified a total of 616 women in same-sex relationships (91.4% of whom self-identified as lesbian or bisexual), 44,564 in different-sex relationships (99.1% of whom self-identified as heterosexual), and 52,709 non-partnered women (96.9% of whom self-identified as heterosexual). We used a different dataset, and our sample was not limited to women. However, by applying those findings to our sample, we would estimate that 33 of the 36 participants in SSRs may self-identify as non-heterosexual, and 1,028 of the 1,037 participants in MSRs may self-identify as heterosexual. Distinguishing participants in SSR from those in MSR can identify non-heterosexual women with a high degree of accuracy (Taylor and Gonzales, 2022). Nevertheless, some people in same-sex relationships may identify as heterosexual. This may be due to the fact that reducing sexual orientation to discrete categories does not fully capture the fluidity and dimensionality of sexual orientation (Savin-Williams and Vrangalova, 2013). Further research is needed to clarify the association between relationship type (SSR/MSR) and sexual orientation and between these two variables and cognitive health.

The SSR sample included in this study may not be representative of broader LGBTQ+ populations. For example, non-heterosexual older adults are more likely to be single and to live alone (Kim and Fredriksen-Goldsen, 2016; Fredriksen-Goldsen et al., 2017), yet all participants in our sample had a spouse, partner, or companion who could speak to their daily functioning and dementia symptoms. Being in a relationship appears to protect against negative health outcomes, both in heterosexual and non-heterosexual people (Solazzo et al., 2020; Taylor and Gonzales, 2022). The SSR group had a median education of 18 years, which is equivalent to a master's degree. Therefore, this group may represent a highly educated sample of older adults, considering that among sexual minority adults across the whole age spectrum (i.e., ≥25 years old), 35–52% report a level of educational attainment at least equal to an undergraduate degree or greater (Mittleman, 2022). Education is considered to be a proxy measure of cognitive reserve (Stern et al., 2020) and is a protective factor against cognitive decline. Last, LGBTQ+ older adults have significant mental health disparities (Newcomb and Mustanski, 2010; Fredriksen-Goldsen et al., 2013a,b; Hoy-Ellis and Fredriksen-Goldsen, 2017). Yet, our SSR sample reported little neuropsychiatric distress. Grouping people into (binary) categories for the sake of research leads to the erasure of inter- and intra-group differences present among LGBTQ+ populations (e.g., fluidity in gender expression, consensual non-monogamy, non-binary sexual and gender experiences). Sexual and gender minority populations have been hard to reach for research purposes (Umberson et al., 2015), so our decision to approximate sexual orientation was driven by a need to demonstrate the challenges present in current AD and related dementia research although our findings may not generalize to the whole population of non-heterosexual older adults.

The limited and heterogeneous availability of data across participants demands caution with comparing results regarding brain structural and cognitive outcome measures. Similarly, the lack of data on other relevant cognitive domains (e.g., response inhibition, visual long-term memory, social cognitive abilities, etc.) leaves open questions about possible differences in other cognitive functions. The lack of variables related to stress and social inequities prevents any definite conclusion on the psychosocial relevance of these results. Data on volumes of subcortical structures, such as the thalamus, basal ganglia, hypothalamus, and amygdala, were also not available. Those regions are essential nodes in brain circuits and are connected to temporal and cingulate areas that have been associated with neuropsychiatric alterations. As such, we cannot exclude the possibility that subcortical nuclei may be differentially affected by sexual minority status. Last, the design of this study was cross-sectional. This hindered our ability to investigate within-subject decline in brain and cognitive health.

### Conclusion and future directions

The findings of this study revealed differential associations between neuropsychiatric alterations and both cognitive performance and brain structure in participants in either SSR or MSR. Those alterations were especially strong for participants with no cognitive impairments, whereas the profiles of the cognitively impaired groups appeared to be very similar. The detrimental impact of neuropsychiatric symptoms on brain structure and cognitive status was also confirmed. However, no striking differences in the outcome measures appeared to be present between SSR and MSR groups, especially in cognitively impaired participants. AD-related neurodegenerative processes may be driving macrostructural brain alterations and cognitive decline to a greater extent than the effects of environmental factors that have been hypothesized to affect cognition in sexual minority older adults (e.g., minority stress; Correro and Nielson, 2019). Within NACC datasets, people in SSR may be more resilient to cognitive decline relative to people in MSR.

Future studies should directly investigate the longitudinal changes in cognition and whole-brain decline, both in structure and function, in sexual minority adults. The assessment of proxy measures of minority stressors experienced by non-heterosexual older adults (e.g., discrimination, microaggressions, internalized homophobia) may help identify people at greater risk of developing cognitive problems. As such, studies need to be designed to collect relevant data (e.g., sexual orientation, non-binary gender identities) that are currently lacking in many epidemiological datasets. The underrepresentation of sexual and gender minority groups in neuroscience research has hindered progress in this field. To date, the impact of minority stress on cognition of non-heterosexual older adults has only been hypothesized but not tested. Examination of specific minority stressors will foster the understanding of stress and resilience mechanisms in LGBTQ+ aging. We anticipate direct and indirect effects will emerge, requiring multivariate and conditional analyses. Therefore, large-scale, longitudinal, prospective studies are needed to better represent LGBTQ+ experiences in human neuroscience with the integration of advanced analytical approaches.

Newer, queerer psychosocial models may assist in understanding protection from dementia broadly and to understand resilience in LGBTQ+ populations specifically. Nicholson et al. (2022) presented a minority mosaic framework for neuroimaging research, emphasizing complex relationships between/within sociocultural and individual factors. We agree that a strictly biological, deterministic perspective cannot appropriately or comprehensively capture unique individual experiences.

## Data availability statement

Publicly available datasets were analyzed in this study. These data can be found at: https://naccdata.org/.

## Ethics statement

Ethical review and approval was not required for the study on human participants in accordance with the local legislation and institutional requirements. The patients/participants provided their written informed consent to participate in this study.

## Author contributions

RM designed this study, selected the dataset, conducted the statistical analyses, contributed to interpreting the findings, and wrote this manuscript. AC conceived this study, contributed to interpreting findings, and wrote this manuscript. KG conceived this study, obtained the datasets, contributed to interpreting the findings, and assisted in manuscript preparation. JF conceived this study, contributed to interpreting the findings, and assisted in manuscript preparation. All authors approved the final version of this manuscript.

## Funding

This work was supported in part by grants K01AG056669 and R24AG066599 (JF). The NACC database was funded by NIA/NIH Grant U24 AG072122. NACC data are contributed by the NIA-funded ADRCs: P30 AG019610 (PI Eric Reiman, MD), P30 AG013846 (PI Neil Kowall, MD), P50 AG008702 (PI Scott Small, MD), P50 AG025688 (PI Allan Levey, MD, PhD), P50 AG047266 (PI Todd Golde, MD, PhD), P30 AG010133 (PI Andrew Saykin, PsyD), P50 AG005146 (PI Marilyn Albert, PhD), P50 AG005134 (PI Bradley Hyman, MD, PhD), P50 AG016574 (PI Ronald Petersen, MD, PhD), P50 AG005138 (PI Mary Sano, PhD), P30 AG008051 (PI Thomas Wisniewski, MD), P30 AG013854 (PI Robert Vassar, PhD), P30 AG008017 (PI Jeffrey Kaye, MD), P30 AG010161 (PI David Bennett, MD), P50 AG047366 (PI Victor Henderson, MD, MS), P30 AG010129 (PI Charles DeCarli, MD), P50 AG016573 (PI Frank LaFerla, PhD), P50 AG005131 (PI James Brewer, MD, PhD), P50 AG023501 (PI Bruce Miller, MD), P30 AG035982 (PI Russell Swerdlow, MD), P30 AG028383 (PI Linda Van Eldik, PhD), P30 AG053760 (PI Henry Paulson, MD, PhD), P30 AG010124 (PI John Trojanowski, MD, PhD), P50 AG005133 (PI Oscar Lopez, MD), P50 AG005142 (PI Helena Chui, MD), P30 AG012300 (PI Roger Rosenberg, MD), P30 AG049638 (PI Suzanne Craft, PhD), P50 AG005136 (PI Thomas Grabowski, MD), P50 AG033514 (PI Sanjay Asthana, MD, FRCP), P50 AG005681 (PI John Morris, MD), and P50 AG047270 (PI Stephen Strittmatter, MD, PhD).

## Conflict of interest

The authors declare that the research was conducted in the absence of any commercial or financial relationships that could be construed as a potential conflict of interest.

## Publisher's note

All claims expressed in this article are solely those of the authors and do not necessarily represent those of their affiliated organizations, or those of the publisher, the editors and the reviewers. Any product that may be evaluated in this article, or claim that may be made by its manufacturer, is not guaranteed or endorsed by the publisher.
