# Supplementary material for: Divergent patterns of cognitive deficits and structural brain alterations between older adults in mixed-sex and same-sex relationships
Source: Front Hum Neurosci. 2022 Sep 2;16:909868. doi: 10.3389/fnhum.2022.909868 (PMC9479099; doi:10.3389/fnhum.2022.909868)
Supplement: Supplementary file 1 [file Data_Sheet_1.docx]

Supplementary Material

**Table S1.** List of gray matter regions of interest by cerebral lobe.

| **Frontal cortices** | **Parietal cortices** | **Occipital cortices** | **Temporal cortices** |
| --- | --- | --- | --- |
| Precentral gyrus | Precuneus | Cuneus | Entorhinal |
| IFG - Pars opercularis | Posterior cingulate | Pericalcarine | Hippocampus |
| IFG - Pars orbitalis | Isthmus cingulate | Lateral occipital | Parahippocampal gyrus |
| IFG - Pars triangularis | Supramarginal gyrus |  | Inferior temporal gyrus |
| Caudal MFG | Inferior parietal |  | Middle temporal gyrus |
| Rostral MFG | Superior parietal |  | Superior temporal gyrus |
| Superior frontal gyrus | Paracentral lobule |  | Transverse temporal |
| Caudal anterior cingulate | Postcentral gyrus |  | Fusiform gyrus |
| Rostral anterior cingulate | Insula |  | Lingual gyrus |
| Lateral orbitofrontal |  |  |  |
| Medial orbitofrontal |  |  |  |

IFG: Inferior frontal gyrus, MFG: Middle frontal gyrus

**Table S2.** Demographic, clinical, and neural characteristics of the sub-sample with available cognitive tests. Values are medians (interquartile range) analyzed with Mann-Whitney *U* test, unless otherwise specified.

| **Variable** | **SSR (*n* = 31)** | **MSR (*n* = 908)** | ***U*** | ***p*** |
| --- | --- | --- | --- | --- |
| Age | 77.00 (15) | 76.00 (13) | 0.96 | 0.336 |
| Education | 18.00 (4) | 16.00 (4) | 1.30 | 0.195 |
| Sex (F/M) | 20/11^a^ | 398/510^a^ | 5.19^b^ | 0.023 |
| ApoE (ε4+/ε4-) | 10/21^a^ | 389/519^a^ | 1.37^b^ | 0.241 |
| Diagnosis (CU/CI) | 22/9^a^ | 494/414^a^ | 3.32^b^ | 0.068 |
| UDS version (1/2/3) | 2/22/7^a^ | 97/613/198^a^ | 0.57^b^ | 0.752 |
| CDR | 0.00 (0.5) | 0.00 (0.5) | -1.80 | 0.072 |
| GMV (cm^3^) | 504.03 (76.26) | 498.59 (74.84) | 0.25 | 0.803 |
| WMV (cm^3^) | 412.61 (79.41) | 410.42 (81.61) | -0.53 | 0.594 |
| CSFV (cm^3^) | 283.50 (83.98) | 297.59 (74.08) | -1.10 | 0.273 |
| TIV (cm^3^) | 1205.13 (198.17) | 1212.80 (183.21) | -0.85 | 0.396 |

CDR: Clinical Dementia Rating scale, CI: Cognitively impaired, CSFV: Cerebrospinal fluid volume, CU: Cognitively unimpaired, GMV: Gray matter volume, TIV: Total intracranial volume, UDS: Uniform Data Set, WMV: White matter volume

^a^ Frequency

^b^ χ^2^

**Table S3.** Significant diagnosis-by-relationship effects on regional brain volumes (Bonferroni-corrected significance *p* < 0.0016). Values are means (standard deviations).

| **Brain regions** | **SSR-CU** | **SSR-CI** | **MSR-CU** | **MSR-CI** | ***F*** | ***p*** |
| --- | --- | --- | --- | --- | --- | --- |
| Left PHG | 3.78 ± 0.56 | 4.05 ± 0.41 | 4.02 ± 0.45 | 3.78 ± 0.53 | 10.22 | 0.001 |

PHG: Parahippocampal gyrus

**Table S4.** Summary statistics for regional gray matter volumes by relationship, sex, and diagnosis. Values are means (standard deviations).

| **Variable** | **MSR** | **SSR** | **Males** | **Females** | **CU** | **CI** |
| --- | --- | --- | --- | --- | --- | --- |
| *Unadjusted values* | | | | | | |
| L HIP | 3.1 (0.5) | 3.1 (0.4) | 3.1 (0.5) | 3.0 (0.4) | 3.3 (0.4) | 2.8 (0.5) |
| L cACC | 3.4 (0.8) | 3.5 (0.8) | 3.6 (0.8) | 3.2 (0.7) | 3.4 (0.8) | 3.4 (0.8) |
| L cMFG | 4.7 (1.0) | 4.8 (1.1) | 4.9 (1.1) | 4.5 (0.9) | 4.9 (1.0) | 4.5 (1.0) |
| L cuneus | 4.4 (1.0) | 4.3 (0.8) | 4.7 (1.0) | 4.1 (0.8) | 4.4. (0.9) | 4.4 (1.0) |
| L EC | 4.1 (0.7) | 4.2 (0.6) | 4.3 (0.7) | 3.8 (0.6) | 4.3 (0.6) | 3.9 (0.8) |
| L FG | 9.3 (1.5) | 9.4 (1.3) | 9.7 (1.5) | 8.9 (1.3) | 9.6 (1.3) | 9.1 (1.6) |
| L IPL | 11.3 (2.0) | 11.3 (1.9) | 11.7 (2.0) | 10.9 (2.0) | 11.7 (2.0) | 10.9 (2.0) |
| L ITG | 9.5 (1.6) | 9.6 (1.6) | 9.9 (1.6) | 8.9 (1.4) | 9.8 (1.4) | 9.0 (1.6) |
| L IC | 6.1 (0.8) | 6.1 (0.9) | 6.3 (0.8) | 5.8 (0.7) | 6.2 (0.7) | 5.9 (0.8) |
| L ICC | 2.7 (0.5) | 2.8 (0.5) | 2.9 (0.5) | 2.5 (0.4) | 2.8 (0.5) | 2.7 (0.6) |
| L lOC | 10.5 (2.0) | 10.1 (1.2) | 11.0 (2.1) | 9.9 (1.7) | 10.8 (1.8) | 10.2 (2.0) |
| L lOFC | 8.1 (1.0) | 8.2 (1.0) | 8.4 (1.1) | 7.7 (0.9) | 8.3 (1.0) | 7.9 (1.1) |
| L LG | 6.6 (1.2) | 6.7 (1.1) | 7.0 (1.2) | 6.2 (1.0) | 6.6. (1.1) | 6.6 (1.3) |
| L mOFC | 4.4 (0.7) | 4.4 (0.9) | 4.6 (0.7) | 4.2 (0.6) | 4.5 (0.7) | 4.3 (0.7) |
| L MTG | 12.7 (2.0) | 12.8 (1.9) | 13.3 (2.0) | 12.0 (1.8) | 13.2 (1.9) | 12.1 (2.0) |
| L PCL | 4.2 (0.9) | 4.2 (0.9) | 4.3 (1.0) | 4.1 (0.9) | 4.3 (0.9) | 4.0 (0.9) |
| L PHG | 3.9 (0.5) | 3.9 (0.5) | 4.0 (0.5) | 3.7 (0.5) | 4.0 (0.5) | 3.8 (0.5) |
| L pOP | 4.4 (0.9) | 4.3 (0.8) | 4.6 (0.9) | 4.2 (0.8) | 4.5 (0.9) | 4.3 (0.9) |
| L pOR | 2.1 (0.4) | 2.1 (0.5) | 2.1 (0.5) | 2.0 (0.4) | 2.1 (0.4) | 2.0 (0.4) |
| L pTR | 4.1 (0.9) | 4.1 (0.7) | 4.3 (0.9) | 4.0 (0.8) | 4.2 (0.9) | 4.0 (0.9) |
| L PEC | 2.6 (0.7) | 2.7 (0.7) | 2.7 (0.7) | 2.5 (0.6) | 2.7 (0.6) | 2.6 (0.7) |
| L POG | 9.2 (1.6) | 9.1 (1.5) | 9.4 (1.6) | 8.9 (1.5) | 9.4 (1.6) | 8.9 (1.6) |
| L PCC | 4.2 (0.7) | 4.2 (0.6) | 4.3 (0.7) | 4.0 (0.6) | 4.2 (0.7) | 4.1 (0.7) |
| L PRG | 12.6 (2.3) | 12.3 (2.0) | 13.0 (2.4) | 12.2 (2.2) | 13.0 (2.2) | 12.1 (2.4) |
| L PreC | 9.7 (1.6) | 9.8 (1.4) | 10.1 (1.6) | 9.2 (1.3) | 10.0 (1.5) | 9.3 (1.6) |
| L rACC | 3.7 (0.8) | 3.8 (0.5) | 3.9 (0.7) | 3.5 (0.7) | 3.8 (0.7) | 3.7 (0.8) |
| L rMFG | 10.6 (1.8) | 10.1 (2.0) | 11.0 (1.9) | 10.1 (1.6) | 10.8 (1.7) | 10.4 (1.8) |
| L SFG | 22.8 (3.8) | 22.9 (3.8) | 23.6 (4.0) | 21.8 (3.4) | 23.6 (3.6) | 21.9 (3.8) |
| L SPL | 10.5 (1.8) | 10.6 (1.9) | 10.9 (1.8) | 10.1 (1.7) | 10.8 (1.7) | 10.2 (1.8) |
| L STG | 15.5 (2.1) | 15.4 (1.9) | 16.1 (2.1) | 14.7 (1.9) | 15.9 (2.0) | 14.9 (2.1) |
| L SMG | 11.0 (1.8) | 10.8 (1.7) | 11.4 (1.9) | 10.4 (1.6) | 11.2 (1.8) | 10.7 (1.9) |
| L TT | 0.9 (0.2) | 0.9 (0.2) | 0.9 (0.2) | 0.9 (0.2) | 0.9 (0.2) | 0.9 (0.2) |
| R HIP | 3.1 (0.5) | 3.2 (0.4) | 3.2 (0.5) | 3.0 (0.4) | 3.3 (0.4) | 2.9 (0.5) |
| R cACC | 2.0 (0.6) | 2.0 (0.6) | 2.2 (0.6) | 1.9 (0.5) | 2.0 (0.6) | 2.1 (0.6) |
| R cMFG | 6.5 (1.4) | 6.3 (1.2) | 6.8 (1.4) | 6.0 (1.2) | 6.6 (1.4) | 6.3 (1.4) |
| R cuneus | 4.2 (0.9) | 4.2 (0.7) | 4.4 (0.9) | 4.0 (0.8) | 4.3 (0.8) | 4.1 (0.9) |
| R EC | 3.9 (0.7) | 3.9 (0.6) | 4.1 (0.7) | 3.6 (0.6) | 4.1 (0.6) | 3.7 (0.8) |
| R FG | 7.9 (1.2) | 8.3 (1.1) | 8.2 (1.3) | 7.5 (1.1) | 8.1 (1.2) | 7.7 (1.3) |
| R IPL | 12.4 (2.0) | 12.1 (1.6) | 12.6 (2.2) | 11.9 (1.8) | 12.7 (1.9) | 11.9 (2.1) |
| R ITG | 10.9 (1.7) | 11.2 (1.6) | 11.4 (1.7) | 10.3 (1.5) | 11.3 (1.5) | 10.4 (1.8) |
| R IC | 6.3 (0.8) | 6.4 (0.8) | 6.5 (0.8) | 6.0 (0.7) | 6.4 (0.7) | 6.2 (0.8) |
| R ICC | 2.7 (0.5) | 2.7 (0.4) | 2.9 (0.5) | 2.5 (0.4) | 2.8 (0.5) | 2.7 (0.6) |
| R lOC | 10.5 (1.9) | 10.6 (1.8) | 11.0 (2.0) | 10.0 (1.7) | 10.9 (1.8) | 10.1 (2.0) |
| R lOFC | 8.4 (1.1) | 8.5 (0.9) | 8.7 (1.1) | 8.0 (0.9) | 8.5 (1.0) | 8.2 (1.1) |
| R LG | 7.4 (1.4) | 7.3 (1.1) | 7.8 (1.4) | 6.9 (1.1) | 7.4 (1.3) | 7.4 (1.4) |
| R mOFC | 4.6 (0.8) | 4.6 (0.9) | 4.7 (0.8) | 4.4 (0.7) | 4.7 (0.8) | 4.4 (0.8) |
| R MTG | 12.5 (1.9) | 12.6 (1.8) | 13.0 (1.9) | 11.8 (1.7) | 13.0 (1.8) | 11.9 (2.0) |
| R PCL | 4.1 (0.9) | 4.3 (0.9) | 4.2 (0.9) | 4.1 (0.9) | 4.3 (0.9) | 3.9 (0.9) |
| R PHG | 4.0 (0.5) | 4.1 (0.4) | 4.2 (0.5) | 3.9 (0.5) | 4.2 (0.5) | 3.9 (0.6) |
| R pOP | 4.5 (0.9) | 4.5 (0.8) | 4.6 (0.9) | 4.3 (0.8) | 4.6 (0.8) | 4.4 (0.9) |
| R pOR | 1.9 (0.4) | 1.9 (0.4) | 1.9 (0.4) | 1.8 (0.4) | 1.9 (0.4) | 1.8 (0.4) |
| R pTR | 4.1 (0.9) | 4.1 (0.9) | 4.2 (0.9) | 3.9 (0.8) | 4.2 (0.9) | 4.0 (0.9) |
| R PEC | 2.7 (0.7) | 2.7 (0.5) | 2.7 (0.7) | 2.6 (0.6) | 2.8 (0.6) | 2.5 (0.6) |
| R POG | 9.0 (1.6) | 9.2 (1.3) | 9.3 (1.6) | 8.7 (1.4) | 9.1 (1.6) | 8.8 (1.6) |
| R PCC | 3.9 (0.7) | 3.9 (0.6) | 4.1 (0.7) | 3.7 (0.6) | 4.0 (0.6) | 3.9 (0.7) |
| R PRG | 11.7 (2.0) | 11.8 (1.7) | 12.1 (2.1) | 11.3 (1.8) | 12.0 (1.9) | 11.4 (2.1) |
| R PreC | 9.3 (1.5) | 9.4 (1.4) | 9.7 (1.6) | 8.9 (1.3) | 9.6 (1.4) | 8.9 (1.5) |
| R rACC | 2.5 (0.6) | 2.6 (0.5) | 2.7 (0.6) | 2.3 (0.5) | 2.6 (0.6) | 2.5 (0.6) |
| R rMFG | 10.9 (1.8) | 10.4 (1.5) | 11.3 (1.9) | 10.4 (1.6) | 11.0 (1.8) | 10.7 (1.8) |
| R SFG | 25.2 (4.0) | 25.6 (3.3) | 26.1 (4.1) | 24.1 (3.5) | 26.1 (3.8) | 24.3 (3.9) |
| R SPL | 10.7 (1.8) | 11.0 (1.9) | 11.0 (1.9) | 10.4 (1.7) | 11.1 (1.7) | 10.3 (1.8) |
| R STG | 13.8 (2.0) | 13.7 (1.5) | 14.4 (2.0) | 13.0 (1.7) | 14.1 (1.8) | 13.3 (2.1) |
| R SMG | 8.8 (1.6) | 8.7 (1.4) | 9.2 (1.6) | 8.4 (1.5) | 9.0 (1.5) | 8.6 (1.7) |
| R TT | 0.9 (0.2) | 0.9 (0.2) | 0.9 (0.3) | 0.9 (0.2) | 0.9 (0.3) | 0.8 (0.2) |
| *Adjusted values* | | | | | | |
| L HIP | 3.0 (0.4) | 3.1 (0.4) | 3.1 (0.9) | 3.1 (0.8) | 3.2 (1.0) | 2.9 (1.3) |
| L cACC | 3.4 (0.7) | 3.6 (0.7) | 3.4 (1.7) | 3.6 (1.5) | 3.4 (1.9) | 3.6 (2.3) |
| L cMFG | 4.7 (0.9) | 4.8 (0.9) | 4.7 (2.0) | 4.9 (1.8) | 5.0 (2.2) | 4.6 (2.7) |
| L cuneus | 4.4 (0.9) | 4.4 (0.9) | 4.5 (1.9) | 4.4 (1.8) | 4.4 (2.2) | 4.4 (2.6) |
| L EC | 4.1 (0.6) | 4.2 (0.6) | 4.2 (1.3) | 4.0 (1.2) | 4.2 (1.5) | 4.0 (1.8) |
| L FG | 9.3 (1.3) | 9.6 (1.3) | 9.5 (2.9) | 9.4 (2.6) | 9.6 (3.2) | 9.3 (3.9) |
| L IPL | 11.3 (1.7) | 11.5 (1.8) | 11.3 (4.0) | 11.5 (3.7) | 11.7 (4.4) | 11.1 (5.4) |
| L ITG | 9.4 (1.3) | 9.6 (1.3) | 9.7 (2.9) | 9.3 (2.7) | 9.9 (3.2) | 9.1 (3.9) |
| L IC | 6.1 (0.6) | 6.1 (0.6) | 6.1 (1.4) | 6.1 (1.2) | 6.3 (1.8) | 5.8 (1.1) |
| L ICC | 2.7 (0.5) | 2.8 (0.5) | 2.8 (1.0) | 2.7 (0.9) | 2.9 (1.1) | 2.7 (1.4) |
| L lOC | 10.5 (1.7) | 10.2 (1.8) | 10.4 (3.9) | 10.3 (3.6) | 10.5 (4.4) | 10.2 (5.3) |
| L lOFC | 8.1 (0.8) | 8.2 (0.8) | 8.2 (1.7) | 8.2 (1.6) | 8.4 (1.9) | 7.9 (2.3) |
| L LG | 6.6 (1.0) | 6.8 (1.0) | 6.7 (2.3) | 6.7 (2.1) | 6.6 (2.5) | 6.8 (3.1) |
| L mOFC | 4.4 (0.6) | 4.4 (0.6) | 4.4 (1.3) | 4.4 (1.2) | 4.7 (1.5) | 4.1 (1.8) |
| L MTG | 12.6 (1.5) | 12.8 (1.6) | 12.8 (3.5) | 12.6 (3.2) | 13.3 (3.9) | 12.2 (4.8) |
| L PCL | 4.2 (0.8) | 4.3 (0.8) | 4.1 (1.8) | 4.3 (1.7) | 4.4 (2.1) | 4.0 (2.5) |
| L PHG | 3.9 (0.4) | 3.9 (0.4) | 3.9 (1.0) | 3.9 (0.9) | 3.9 (1.1) | 3.9 (1.3) |
| L pOP | 4.4 (0.8) | 4.3 (0.8) | 4.3 (1.8) | 4.4 (1.6) | 4.6 (2.0) | 4.1 (2.4) |
| L pOR | 2.1 (0.4) | 2.1 (0.4) | 2.1 (0.9) | 2.1 (0.9) | 2.1 (1.1) | 2.0 (1.3) |
| L pTR | 4.1 (0.8) | 4.1 (0.8) | 4.1 (1.9) | 4.2 (1.7) | 4.2 (2.1) | 4.1 (2.6) |
| L PEC | 2.6 (0.6) | 2.8 (0.6) | 2.6 (1.4) | 2.8 (1.3) | 2.6 (1.6) | 2.8 (1.9) |
| L POG | 9.2 (1.3) | 9.1 (1.3) | 8.8 (2.9) | 9.5 (2.7) | 9.3 (3.3) | 9.1 (4.0) |
| L PCC | 4.2 (0.6) | 4.2 (0.6) | 4.2 (1.3) | 4.2 (1.2) | 4.3 (1.5) | 4.1 (1.8) |
| L PRG | 12.7 (1.8) | 12.3 (1.9) | 12.1 (4.2) | 12.9 (3.8) | 13.0 (4.7) | 12.0 (5.7) |
| L PreC | 9.6 (1.2) | 9.8 (1.3) | 9.7 (2.8) | 9.7 (2.6) | 10.1 (3.1) | 9.3 (3.8) |
| L rACC | 3.7 (0.6) | 3.9 (0.7) | 3.8 (1.5) | 3.8 (1.3) | 3.8 (1.6) | 3.8 (2.0) |
| L rMFG | 10.6 (1.5) | 10.2 (1.5) | 10.3 (3.5) | 10.5 (3.2) | 10.6 (3.9) | 10.1 (4.7) |
| L SFG | 22.8 (2.9) | 22.7 (3.0) | 22.4 (6.6) | 23.1 (6.0) | 23.9 (7.4) | 21.6 (8.9) |
| L SPL | 10.5 (1.5) | 10.5 (1.5) | 10.4 (3.4) | 10.7 (3.1) | 10.9 (3.8) | 10.1 (4.6) |
| L STG | 15.4 (1.6) | 15.4 (1.6) | 15.5 (3.5) | 15.4 (3.3) | 15.9 (4.0) | 15.0 (4.8) |
| L SMG | 11.0 (1.5) | 10.8 (1.5) | 10.7 (3.3) | 11.0 (3.0) | 11.3 (3.7) | 10.5 (4.5) |
| L TT | 0.9 (0.2) | 0.9 (0.2) | 0.9 (0.5) | 0.9 (0.4) | 1.0 (0.5) | 0.9 (0.7) |
| R HIP | 3.1 (0.4) | 3.2 (0.4) | 3.1 (0.9) | 3.2 (0.9) | 3.3 (1.0) | 3.0 (1.2) |
| R cACC | 2.1 (0.6) | 2.1 (0.6) | 2.1 (1.3) | 2.1 (1.2) | 2.0 (1.4) | 2.2 (1.7) |
| R cMFG | 6.5 (1.1) | 6.4 (1.1) | 6.4 (2.5) | 6.5 (2.3) | 6.6 (2.8) | 6.3 (3.4) |
| R cuneus | 4.2 (0.8) | 4.3 (0.8) | 4.2 (1.7) | 4.2 (1.6) | 4.3 (1.9) | 4.2 (2.3) |
| R EC | 3.9 (0.6) | 3.9 (0.6) | 4.0 (1.4) | 3.8 (1.3) | 4.0 (1.6) | 3.7 (1.9) |
| R FG | 7.9 (1.0) | 8.3 (1.1) | 8.1 (2.4) | 8.1 (2.2) | 8.3 (2.6) | 8.0 (3.2) |
| R IPL | 12.4 (1.8) | 12.2 (1.8) | 12.2 (4.0) | 12.4 (3.7) | 12.6 (4.5) | 12.0 (5.4) |
| R ITG | 10.8 (1.4) | 11.2 (1.4) | 11.2 (3.1) | 10.8 (2.9) | 11.5 (3.5) | 10.5 (4.2) |
| R IC | 6.3 (0.6) | 6.4 (0.6) | 6.3 (1.3) | 6.3 (1.2) | 6.5 (1.5) | 6.2 (1.8) |
| R ICC | 2.7 (0.4) | 2.7 (0.4) | 2.8 (1.0) | 2.7 (0.9) | 2.8 (1.1) | 2.7 (1.3) |
| R lOC | 10.5 (1.7) | 10.7 (1.7) | 10.6 (3.8) | 10.6 (3.5) | 10.7 (4.2) | 10.4 (5.1) |
| R lOFC | 8.4 (0.8) | 8.6 (0.8) | 8.5 (1.9) | 8.5 (1.7) | 8.6 (2.1) | 8.4 (2.5) |
| R LG | 7.4 (1.1) | 7.5 (1.1) | 7.5 (2.5) | 7.4 (2.3) | 7.4 (2.8) | 7.5 (3.5) |
| R mOFC | 4.6 (0.6) | 4.5 (0.6) | 4.5 (1.4) | 4.6 (1.3) | 4.8 (1.6) | 4.3 (1.9) |
| R MTG | 12.4 (1.5) | 12.7 (1.5) | 12.6 (3.3) | 12.6 (3.1) | 13.0 (3.7) | 12.1 (4.5) |
| R PCL | 4.2 (0.8) | 4.3 (0.8) | 4.1 (1.8) | 4.4 (1.6) | 4.4 (2.0) | 4.1 (2.4) |
| R PHG | 4.0 (0.4) | 4.1 (0.4) | 4.1 (1.0) | 4.1 (0.9) | 4.1 (1.1) | 4.0 (1.3) |
| R pOP | 4.5 (0.8) | 4.6 (0.8) | 4.5 (1.8) | 4.6 (1.6) | 4.5 (2.0) | 4.6 (2.4) |
| R pOR | 1.9 (0.4) | 1.9 (0.4) | 1.9 (0.9) | 1.9 (0.8) | 1.9 (1.0) | 1.9 (1.2) |
| R pTR | 4.1 (0.8) | 4.1 (0.8) | 4.0 (1.9) | 4.2 (1.7) | 4.2 (2.1) | 4.0 (2.6) |
| R PEC | 2.7 (0.6) | 2.8 (0.6) | 2.6 (1.3) | 2.8 (1.2) | 2.7 (1.5) | 2.7 (1.8) |
| R POG | 9.0 (1.3) | 9.3 (1.3) | 8.9 (2.9) | 9.4 (2.7) | 9.2 (3.2) | 9.2 (3.9) |
| R PCC | 3.9 (0.5) | 4.0 (0.6) | 3.9 (1.2) | 4.0 (1.1) | 4.0 (1.4) | 3.9 (1.7) |
| R PRG | 11.7 (1.6) | 11.9 (1.6) | 11.5 (3.6) | 12.1 (3.3) | 12.1 (4.0) | 11.5 (4.8) |
| R PreC | 9.3 (1.2) | 9.4 (1.2) | 9.3 (2.7) | 9.4 (2.5) | 9.8 (3.0) | 8.9 (3.6) |
| R rACC | 2.5 (0.5) | 2.6 (0.5) | 2.6 (1.1) | 2.6 (1.0) | 2.6 (1.3) | 2.6 (1.5) |
| R rMFG | 10.9 (1.5) | 10.5 (1.5) | 10.5 (3.4) | 10.8 (3.1) | 10.8 (3.8) | 10.5 (4.6) |
| R SFG | 25.2 (2.9) | 25.6 (3.0) | 25.0 (6.6) | 25.8 (6.1) | 26.4 (7.3) | 24.5 (9.0) |
| R SPL | 10.7 (1.5) | 11.1 (1.6) | 10.6 (3.5) | 11.1 (3.2) | 11.2 (3.9) | 10.6 (4.8) |
| R STG | 13.7 (1.5) | 13.8 (1.5) | 13.8 (3.3) | 13.7 (3.1) | 14.1 (3.7) | 13.4 (4.5) |
| R SMG | 8.8 (1.4) | 8.8 (1.4) | 8.7 (3.1) | 8.9 (2.9) | 8.9 (3.5) | 8.7 (4.2) |
| R TT | 0.9 (0.2) | 0.9 (0.2) | 0.8 (0.5) | 0.9 (0.5) | 0.9 (0.6) | 0.8 (0.7) |

cACC: Caudal anterior cingulate cortex, cMFG: Caudal middle frontal gyrus, EC: Entorhinal cortex, FG: Fusiform gyrus, HIP: Hippocampus, IC: Insular cortex, ICC: Isthmus cingulate cortex, IPL: Inferior parietal lobule, ITG: Inferior temporal gyrus, L: Left, LG: Lingual gyrus, lOC: Lateral occipital cortex, lOFC: Lateral orbitofrontal cortex, mOFC: Medial orbitofrontal cortex, MTG: Middle temporal gyrus, PCC: Posterior cingulate, PCL: Paracentral lobule, PEC: Pericalcarine cortex, PHG: Parahippocampal gyrus, POG: Postcentral gyrus, pOP: Pars opercularis, pOR: Pars orbitalis, PreC: Precuneus, PRG: Precentral gyrus, pTR: Pars triangularis, R: Right, rACC: Rostral anterior cingulate, rMFG: Rostral middle frontal gyrus, SFG: Superior frontal gyrus, SMG: Supramarginal gyrus, SPL: Superior parietal lobule, STG: Superior temporal gyrus, TT: Transverse temporal

**Table S5.** Summary statistics for regional cortical thickness by relationship, sex, and diagnosis. Values are means (standard deviations).

| **Variable** | **MSR** | **SSR** | **Males** | **Females** | **CU** | **CI** |
| --- | --- | --- | --- | --- | --- | --- |
| *Unadjusted values* | | | | | | |
| L cACC | 2.5 (0.4) | 2.6 (0.5) | 2.4 (0.4) | 2.5 (0.4) | 2.6 (0.4) | 2.4 (0.4) |
| L cMFG | 1.9 (0.4) | 1.9 (0.4) | 1.8 (0.4) | 1.9 (0.4) | 2.0 (0.3) | 1.8 (0.5) |
| L cuneus | 1.3 (0.3) | 1.3 (0.2) | 1.3 (0.3) | 1.3 (0.2) | 1.3 (0.2) | 1.3 (0.4) |
| L EC | 3.3 (0.7) | 3.4 (0.6) | 3.3 (0.7) | 3.4 (0.7) | 3.6 (0.5) | 3.0 (0.7) |
| L FG | 2.3 (0.4) | 2.3 (0.4) | 2.3 (0.4) | 2.4 (0.4) | 2.4 (0.4) | 2.3 (0.5) |
| L IPL | 2.0 (0.3) | 2.0 (0.4) | 2.0 (0.3) | 2.1 (0.3) | 2.1 (0.3) | 2.0 (0.4) |
| L ITG | 2.6 (0.5) | 2.6 (0.5) | 2.6 (0.5) | 2.6 (0.5) | 2.7 (0.5) | 2.5 (0.5) |
| L IC | 3.2 (0.4) | 3.4 (0.6) | 3.2 (0.5) | 3.2 (0.4) | 3.3 (0.4) | 3.1 (0.5) |
| L ICC | 2.2 (0.4) | 2.3 (0.3) | 2.2 (0.4) | 2.2 (0.3) | 2.3 (0.3) | 2.1 (0.4) |
| L lOC | 1.7 (0.4) | 1.6 (0.3) | 1.7 (0.4) | 1.7 (0.3) | 1.7 (0.3) | 1.7 (0.4) |
| L lOFC | 2.3 (0.3) | 2.4 (0.3) | 2.3 (0.3) | 2.3 (0.3) | 2.4 (0.3) | 2.2 (0.4) |
| L LG | 1.6 (0.4) | 1.6 (0.3) | 1.6 (0.4) | 1.6 (0.4) | 1.6 (0.3) | 1.5 (0.5) |
| L mOFC | 2.3 (0.3) | 2.4 (0.3) | 2.3 (0.3) | 2.3 (0.3) | 2.4 (0.3) | 2.2 (0.4) |
| L MTG | 2.4 (0.4) | 2.4 (0.4) | 2.4 (0.4) | 2.4 (0.4) | 2.5 (0.3) | 2.3 (0.4) |
| L PCL | 1.6 (0.4) | 1.7 (0.3) | 1.5 (0.4) | 1.6 (0.4) | 1.7 (0.3) | 1.5 (0.4) |
| L PHG | 1.8 (0.3) | 1.8 (0.3) | 1.8 (0.3) | 1.8 (0.3) | 1.9 (0.3) | 1.7 (0.3) |
| L pOP | 1.9 (0.3) | 1.9 (0.3) | 1.9 (0.3) | 1.9 (0.3) | 1.9 (0.2) | 1.8 (0.3) |
| L pOR | 2.0 (0.3) | 2.0 (0.4) | 2.0 (0.3) | 2.0 (0.3) | 2.1 (0.3) | 2.0 (0.4) |
| L pTR | 1.9 (0.3) | 1.8 (0.3) | 1.9 (0.3) | 1.9 (0.3) | 1.9 (0.2) | 1.8 (0.3) |
| L PEC | 1.1 (0.4) | 1.1 (0.3) | 1.1 (0.4) | 1.1 (0.3) | 1.1 (0.3) | 1.0 (0.4) |
| L POG | 1.4 (0.2) | 1.4 (0.2) | 1.4 (0.3) | 1.4 (0.2) | 1.5 (0.2) | 1.4 (0.3) |
| L PCC | 2.2 (0.3) | 2.3 (0.3) | 2.2 (0.3) | 2.3 (0.3) | 2.3 (0.3) | 2.1 (0.3) |
| L PRG | 1.6 (0.3) | 1.6 (0.3) | 1.6 (0.3) | 1.7 (0.3) | 1.7 (0.3) | 1.5 (0.4) |
| L PreC | 1.9 (0.3) | 1.9 (0.2) | 1.9 (0.3) | 1.9 (0.3) | 1.9 (0.2) | 1.8 (0.4) |
| L rACC | 3.0 (0.5) | 3.1 (0.5) | 2.9 (0.5) | 3.0 (0.4) | 3.1 (0.4) | 2.8 (0.5) |
| L rMFG | 2.1 (0.4) | 2.0 (0.4) | 2.1 (0.4) | 2.1 (0.3) | 2.1 (0.3) | 2.1 (0.4) |
| L SFG | 2.1 (0.4) | 2.2 (0.3) | 2.1 (0.4) | 2.2 (0.3) | 2.2 (0.3) | 2.0 (0.4) |
| L SPL | 1.6 (0.3) | 1.6 (0.3) | 1.6 (0.3) | 1.6 (0.2) | 1.6 (0.2) | 1.5 (0.3) |
| L STG | 2.1 (0.3) | 2.1 (0.3) | 2.1 (0.3) | 2.0 (0.3) | 2.1 (0.2) | 2.0 (0.3) |
| L SMG | 2.0 (0.3) | 2.0 (0.3) | 2.0 (0.3) | 2.0 (0.3) | 2.0 (0.2) | 2.0 (0.3) |
| L TT | 1.5 (0.4) | 1.5 (0.4) | 1.4 (0.3) | 1.5 (0.4) | 1.6 (0.3) | 1.4 (0.4) |
| R cACC | 2.7 (0.4) | 2.8 (0.4) | 2.7 (0.4) | 2.7 (0.4) | 2.7 (0.4) | 2.6 (0.5) |
| R cMFG | 1.9 (0.4) | 2.0 (0.3) | 1.9 (0.4) | 1.9 (0.3) | 2.0 (0.3) | 1.9 (0.4) |
| R cuneus | 1.4 (0.3) | 1.4 (0.2) | 1.4 (0.3) | 1.4 (0.3) | 1.4 (0.2) | 1.3 (0.3) |
| R EC | 3.4 (0.7) | 3.5 (0.7) | 3.4 (0.8) | 3.5 (0.7) | 3.7 (0.5) | 3.1 (0.8) |
| R FG | 2.4 (0.5) | 2.4 (0.4) | 2.4 (0.5) | 2.4 (0.5) | 2.5 (0.4) | 2.3 (0.5) |
| R IPL | 1.9 (0.3) | 1.9 (0.2) | 1.9 (0.3) | 1.9 (0.3) | 1.9 (0.3) | 1.9 (0.3) |
| R ITG | 2.6 (0.5) | 2.6 (0.5) | 2.6 (0.5) | 2.7 (0.5) | 2.7 (0.5) | 2.5 (0.5) |
| R IC | 3.2 (0.4) | 3.4 (0.5) | 3.2 (0.4) | 3.2 (0.4) | 3.3 (0.4) | 3.1 (0.5) |
| R ICC | 2.2 (0.4) | 2.3 (0.3) | 2.2 (0.4) | 2.3 (0.3) | 2.4 (0.3) | 2.1 (0.4) |
| R lOC | 1.8 (0.4) | 1.7 (0.3) | 1.8 (0.4) | 1.8 (0.4) | 1.8 (0.3) | 1.7 (0.4) |
| R lOFC | 2.3 (0.4) | 2.4 (0.4) | 2.3 (0.4) | 2.3 (0.4) | 2.4 (0.4) | 2.3 (0.4) |
| R LG | 1.7 (0.4) | 1.7 (0.4) | 1.6 (0.4) | 1.7 (0.4) | 1.7 (0.3) | 1.6 (0.4) |
| R mOFC | 2.3 (0.4) | 2.3 (0.3) | 2.2 (0.4) | 2.3 (0.4) | 2.3 (0.3) | 2.2 (0.4) |
| R MTG | 2.4 (0.4) | 2.4 (0.3) | 2.4 (0.4) | 2.4 (0.4) | 2.4 (0.4) | 2.3 (0.4) |
| R PCL | 1.6 (0.4) | 1.7 (0.3) | 1.5 (0.4) | 1.6 (0.4) | 1.7 (0.3) | 1.5 (0.4) |
| R PHG | 1.9 (0.3) | 2.0 (0.2) | 1.8 (0.3) | 1.9 (0.3) | 1.9 (0.3) | 1.7 (0.4) |
| R pOP | 1.9 (0.3) | 1.9 (0.2) | 1.9 (0.3) | 1.9 (0.3) | 1.9 (0.3) | 1.9 (0.3) |
| R pOR | 2.0 (0.4) | 2.0 (0.4) | 1.9 (0.4) | 2.0 (0.4) | 2.0 (0.4) | 1.9 (0.4) |
| R pTR | 1.9 (0.3) | 1.8 (0.3) | 1.9 (0.4) | 1.8 (0.3) | 1.9 (0.3) | 1.8 (0.4) |
| R PEC | 1.2 (0.4) | 1.2 (0.3) | 1.1 (0.4) | 1.2 (0.3) | 1.2 (0.3) | 1.1 (0.4) |
| R POG | 1.4 (0.2) | 1.4 (0.2) | 1.4 (0.2) | 1.4 (0.2) | 1.5 (0.2) | 1.4 (0.3) |
| R PCC | 2.4 (0.4) | 2.4 (0.4) | 2.4 (0.4) | 2.4 (0.3) | 2.5 (0.3) | 2.3 (0.4) |
| R PRG | 1.6 (0.3) | 1.7 (0.3) | 1.6 (0.3) | 1.6 (0.3) | 1.7 (0.3) | 1.6 (0.3) |
| R PreC | 1.9 (0.3) | 2.0 (0.3) | 1.9 (0.3) | 1.9 (0.3) | 1.9 (0.2) | 1.8 (0.3) |
| R rACC | 2.9 (0.5) | 3.0 (0.5) | 2.9 (0.5) | 2.9 (0.5) | 3.0 (0.4) | 2.8 (0.5) |
| R rMFG | 2.1 (0.4) | 2.0 (0.3) | 2.1 (0.4) | 2.1 (0.4) | 2.1 (0.4) | 2.1 (0.4) |
| R SFG | 2.2 (0.3) | 2.2 (0.3) | 2.2 (0.3) | 2.3 (0.3) | 2.3 (0.3) | 2.1 (0.3) |
| R SPL | 1.5 (0.3) | 1.6 (0.3) | 1.5 (0.3) | 1.5 (0.3) | 1.5 (0.2) | 1.4 (0.3) |
| R STG | 2.1 (0.3) | 2.2 (0.3) | 2.1 (0.4) | 2.1 (0.3) | 2.2 (0.3) | 2.0 (0.4) |
| R SMG | 1.8 (0.3) | 1.8 (0.3) | 1.8 (0.3) | 1.8 (0.3) | 1.9 (0.2) | 1.8 (0.3) |
| R TT | 1.5 (0.4) | 1.6 (0.3) | 1.4 (0.4) | 1.5 (0.3) | 1.5 (0.3) | 1.4 (0.4) |
| *Adjusted values* | | | | | | |
| L cACC | 2.5 (0.4) | 2.5 (0.4) | 2.5 (0.9) | 2.5 (0.9) | 2.6 (1.1) | 2.4 (1.3) |
| L cMFG | 1.9 (0.4) | 1.9 (0.4) | 1.8 (0.9) | 2.0 (0.8) | 2.0 (1.0) | 1.8 (1.2) |
| L cuneus | 1.3 (0.4) | 1.3 (0.3) | 1.3 (0.7) | 1.3 (0.6) | 1.3 (0.8) | 1.3 (0.9) |
| L EC | 3.3 (0.6) | 3.3 (0.7) | 3.3 (1.5) | 3.3 (1.3) | 3.5 (1.6) | 3.1 (2.0) |
| L FG | 2.3 (0.4) | 2.3 (0.4) | 2.3 (1.0) | 2.3 (0.9) | 2.4 (1.1) | 2.3 (1.3) |
| L IPL | 2.0 (0.3) | 2.0 (0.3) | 2.0 (0.7) | 2.0 (0.7) | 2.0 (0.8) | 2.0 (1.0) |
| L ITG | 2.6 (0.5) | 2.5 (0.5) | 2.5 (1.1) | 2.5 (1.0) | 2.6 (1.3) | 2.4 (1.5) |
| L IC | 3.2 (0.4) | 3.3 (0.5) | 3.2 (1.0) | 3.2 (0.9) | 3.4 (1.1) | 3.1 (1.3) |
| L ICC | 2.2 (0.3) | 2.3 (0.3) | 2.2 (0.8) | 2.2 (0.7) | 2.3 (0.9) | 2.1 (1.1) |
| L lOC | 1.7 (0.4) | 1.6 (0.4) | 1.7 (0.8) | 1.7 (0.8) | 1.7 (0.9) | 1.6 (1.1) |
| L lOFC | 2.3 (0.3) | 2.3 (0.3) | 2.3 (0.8) | 2.3 (0.7) | 2.4 (0.8) | 2.2 (1.0) |
| L LG | 1.6 (0.4) | 1.6 (0.4) | 1.6 (0.9) | 1.6 (0.9) | 1.6 (1.0) | 1.6 (1.3) |
| L mOFC | 2.3 (0.3) | 2.4 (0.3) | 2.4 (0.8) | 2.3 (0.7) | 2.4 (0.9) | 2.2 (1.0) |
| L MTG | 2.4 (0.4) | 2.4 (0.4) | 2.4 (0.9) | 2.4 (0.8) | 2.4 (1.0) | 2.3 (1.2) |
| L PCL | 1.6 (0.4) | 1.7 (0.4) | 1.5 (0.9) | 1.7 (0.8) | 1.7 (0.9) | 1.5 (1.2) |
| L PHG | 1.8 (0.3) | 1.8 (0.3) | 1.8 (0.7) | 1.8 (0.6) | 1.8 (0.8) | 1.7 (1.0) |
| L pOP | 1.9 (0.3) | 1.9 (0.3) | 1.9 (0.6) | 1.9 (0.6) | 1.9 (0.7) | 1.8 (0.9) |
| L pOR | 2.0 (0.3) | 1.9 (0.3) | 1.9 (0.8) | 2.0 (0.7) | 2.0 (0.9) | 1.9 (1.1) |
| L pTR | 1.9 (0.3) | 1.8 (0.3) | 1.8 (0.7) | 1.8 (0.6) | 1.9 (0.7) | 1.8 (0.9) |
| L PEC | 1.1 (0.3) | 1.1 (0.4) | 1.0 (0.8) | 1.2 (0.7) | 1.1 (0.9) | 1.1 (1.1) |
| L POG | 1.4 (0.2) | 1.4 (0.2) | 1.4 (0.5) | 1.5 (0.5) | 1.4 (0.6) | 1.4 (0.7) |
| L PCC | 2.2 (0.3) | 2.3 (0.3) | 2.2 (0.7) | 2.2 (0.7) | 2.3 (0.8) | 2.2 (1.0) |
| L PRG | 1.6 (0.3) | 1.6 (0.3) | 1.5 (0.7) | 1.7 (0.7) | 1.7 (0.8) | 1.5 (1.0) |
| L PreC | 1.9 (0.3) | 1.9 (0.3) | 1.9 (0.7) | 1.9 (0.6) | 1.9 (0.7) | 1.8 (0.9) |
| L rACC | 2.9 (0.5) | 3.0 (0.5) | 3.0 (1.0) | 3.0 (1.0) | 3.1 (1.1) | 2.9 (1.4) |
| L rMFG | 2.1 (0.4) | 2.0 (0.4) | 2.0 (0.8) | 2.0 (0.8) | 2.1 (0.9) | 2.0 (1.1) |
| L SFG | 2.1 (0.3) | 2.1 (0.3) | 2.1 (0.8) | 2.2 (0.7) | 2.2 (0.9) | 2.0 (1.1) |
| L SPL | 1.6 (0.3) | 1.6 (0.3) | 1.6 (0.6) | 1.6 (0.6) | 1.6 (0.7) | 1.5 (0.8) |
| L STG | 2.0 (0.3) | 2.0 (0.3) | 2.0 (0.7) | 2.0 (0.6) | 2.1 (0.7) | 2.0 (0.9) |
| L SMG | 2.0 (0.3) | 2.0 (0.3) | 2.0 (0.6) | 2.0 (0.6) | 2.0 (0.7) | 1.9 (0.8) |
| L TT | 1.5 (0.3) | 1.4 (0.3) | 1.4 (0.8) | 1.5 (0.7) | 1.6 (0.8) | 1.3 (1.0) |
| R cACC | 2.7 (0.4) | 2.8 (0.4) | 2.7 (1.0) | 2.7 (0.9) | 2.8 (1.1) | 2.6 (1.3) |
| R cMFG | 1.9 (0.4) | 2.0 (0.4) | 1.9 (0.8) | 2.0 (0.8) | 2.0 (0.9) | 1.9 (1.1) |
| R cuneus | 1.4 (0.3) | 1.4 (0.3) | 1.3 (0.7) | 1.4 (0.6) | 1.4 (0.8) | 1.3 (0.9) |
| R EC | 3.4 (0.7) | 3.4 (0.7) | 3.4 (1.6) | 3.4 (1.4) | 3.7 (1.8) | 3.1 (2.1) |
| R FG | 2.4 (0.5) | 2.4 (0.5) | 2.4 (1.0) | 2.4 (1.0) | 2.5 (1.2) | 2.3 (1.4) |
| R IPL | 1.9 (0.3) | 1.9 (0.3) | 1.9 (0.7) | 1.9 (0.6) | 1.9 (0.8) | 1.9 (1.0) |
| R ITG | 2.6 (0.5) | 2.6 (0.5) | 2.7 (1.2) | 2.6 (1.1) | 2.7 (1.3) | 2.6 (1.6) |
| R IC | 3.2 (0.4) | 3.3 (0.5) | 3.3 (1.0) | 3.3 (0.9) | 3.3 (1.1) | 3.2 (1.4) |
| R ICC | 2.2 (0.4) | 2.3 (0.4) | 2.3 (0.8) | 2.3 (0.7) | 2.4 (0.9) | 2.2 (1.1) |
| R lOC | 1.7 (0.4) | 1.7 (0.4) | 1.7 (0.9) | 1.7 (0.8) | 1.8 (1.0) | 1.7 (1.2) |
| R lOFC | 2.3 (0.4) | 2.4 (0.4) | 2.3 (0.9) | 2.3 (0.8) | 2.4 (1.0) | 2.3 (1.2) |
| R LG | 1.7 (0.4) | 1.7 (0.4) | 1.7 (0.9) | 1.7 (0.8) | 1.8 (1.0) | 1.6 (1.2) |
| R mOFC | 2.2 (0.4) | 2.2 (0.4) | 2.3 (0.8) | 2.2 (0.7) | 2.4 (0.9) | 2.1 (1.1) |
| R MTG | 2.4 (0.4) | 2.4 (0.4) | 2.4 (0.9) | 2.4 (0.8) | 2.4 (1.0) | 2.4 (1.2) |
| R PCL | 1.6 (0.4) | 1.7 (0.4) | 1.5 (0.8) | 1.7 (0.7) | 1.7 (0.9) | 1.5 (1.1) |
| R PHG | 1.8 (0.3) | 1.9 (0.3) | 1.9 (0.7) | 1.9 (0.7) | 2.0 (0.8) | 1.8 (1.0) |
| R pOP | 1.9 (0.3) | 1.9 (0.3) | 1.9 (0.6) | 1.9 (0.6) | 1.9 (0.7) | 1.9 (0.9) |
| R pOR | 1.9 (0.4) | 2.0 (0.4) | 2.0 (0.9) | 2.0 (0.8) | 2.0 (1.0) | 1.9 (1.2) |
| R pTR | 1.9 (0.3) | 1.8 (0.4) | 1.8 (0.8) | 1.8 (0.7) | 1.8 (0.9) | 1.8 (1.1) |
| R PEC | 1.2 (0.4) | 1.2 (0.4) | 1.1 (0.8) | 1.3 (0.8) | 1.2 (0.9) | 1.1 (1.1) |
| R POG | 1.4 (0.2) | 1.4 (0.2) | 1.4 (0.5) | 1.4 (0.5) | 1.4 (0.6) | 1.4 (0.7) |
| R PCC | 2.4 (0.3) | 2.4 (0.4) | 2.4 (0.8) | 2.4 (0.7) | 2.5 (0.9) | 2.3 (1.1) |
| R PRG | 1.6 (0.3) | 1.7 (0.3) | 1.6 (0.7) | 1.7 (0.6) | 1.7 (0.8) | 1.6 (0.9) |
| R PreC | 1.9 (0.3) | 2.0 (0.3) | 1.9 (0.6) | 1.9 (0.6) | 2.0 (0.7) | 1.9 (0.9) |
| R rACC | 2.9 (0.5) | 3.0 (0.5) | 3.0 (1.1) | 2.9 (1.0) | 3.1 (1.2) | 2.8 (1.5) |
| R rMFG | 2.1 (0.4) | 2.0 (0.4) | 2.1 (0.9) | 2.0 (0.8) | 2.0 (1.0) | 2.0 (1.2) |
| R SFG | 2.2 (0.3) | 2.2 (0.3) | 2.2 (0.7) | 2.2 (0.7) | 2.2 (0.8) | 2.1 (1.0) |
| R SPL | 1.5 (0.3) | 1.5 (0.3) | 1.5 (0.6) | 1.5 (0.6) | 1.5 (0.7) | 1.5 (0.9) |
| R STG | 2.1 (0.3) | 2.1 (0.3) | 2.1 (0.8) | 2.1 (0.7) | 2.2 (0.9) | 2.0 (1.1) |
| R SMG | 1.8 (0.3) | 1.8 (0.3) | 1.8 (0.6) | 1.8 (0.6) | 1.8 (0.7) | 1.8 (0.9) |
| R TT | 1.5 (0.4) | 1.5 (0.4) | 1.4 (0.8) | 1.6 (0.7) | 1.6 (0.9) | 1.5 (1.1) |

cACC: Caudal anterior cingulate cortex, cMFG: Caudal middle frontal gyrus, EC: Entorhinal cortex, FG: Fusiform gyrus, IC: Insular cortex, ICC: Isthmus cingulate cortex, IPL: Inferior parietal lobule, ITG: Inferior temporal gyrus, L: Left, LG: Lingual gyrus, lOC: Lateral occipital cortex, lOFC: Lateral orbitofrontal cortex, mOFC: Medial orbitofrontal cortex, MTG: Middle temporal gyrus, PCC: Posterior cingulate, PCL: Paracentral lobule, PEC: Pericalcarine cortex, PHG: Parahippocampal gyrus, POG: Postcentral gyrus, pOP: Pars opercularis, pOR: Pars orbitalis, PreC: Precuneus, PRG: Precentral gyrus, pTR: Pars triangularis, R: Right, rACC: Rostral anterior cingulate, rMFG: Rostral middle frontal gyrus, SFG: Superior frontal gyrus, SMG: Supramarginal gyrus, SPL: Superior parietal lobule, STG: Superior temporal gyrus, TT: Transverse temporal

**Table S6.** Summary statistics for all cognitive variables by relationship, sex, and diagnosis. Values are means (standard deviations).

| **Variable** | **MSR** | **SSR** | **Males** | **Females** | **CU** | **CI** | |
| --- | --- | --- | --- | --- | --- | --- | --- |
| *Unadjusted values* | | | | | | |  |
| MMSE | 26.7 (4.4) | 28.0 (2.7) | 26.2 (4.7) | 27.5 (3.9) | 29.1 (1.3) | 24.0 (5.1) | |
| LMI | 12.1 (6.0) | 15.7 (5.7) | 12.2 (6.0) | 12.2 (6.1) | 12.5 (6.1) | 11.8 (6.1) | |
| LMD | 10.7 (6.6) | 14.4 (6.3) | 10.7 (6.5) | 10.8 (6.7) | 11.0 (6.6) | 10.5 (6.5) | |
| DSF | 6.4 (1.4) | 7.0 (1.4) | 6.5 (1.4) | 6.5 (1.4) | 6.5 (1.4) | 6.4 (1.4) | |
| DSB | 4.5 (1.6) | 5.1 (1.5) | 4.6 (1.6) | 4.5 (1.5) | 4.6 (1.5) | 4.5 (1.6) | |
| SFA | 17.2 (7.5) | 19.9 (7.6) | 17.3 (7.7) | 17.2 (7.3) | 17.6 (7.6) | 16.9 (7.3) | |
| SFV | 11.9 (5.3) | 13.7 (5.7) | 11.9 (5.3) | 12.0 (5.3) | 12.1 (5.3) | 11.7 (5.3) | |
| TMTA | 44.7 (28.3) | 38.5 (26.3) | 44.1 (28.0) | 44.9 (28.5) | 42.7 (26.6) | 46.7 (30.0) | |
| TMTB | 125.0 (81.6) | 87.1 (40.4) | 123.5 (79.6) | 123.8 (82.4) | 120.0 (80.6) | 128.3 (80.9) | |
| *Adjusted values* | | | | | | |  |
| MMSE | 26.5 (3.6) | 27.0 (3.9) | 26.4 (8.7) | 27.1 (7.8) | 28.7 (8.8) | 24.8 (12.2) | |
| LMI | 12.1 (6.2) | 15.7 (6.6) | 13.9 (14.9) | 13.9 (13.3) | 14.2 (15.4) | 13.6 (20.7) | |
| LMD | 10.7 (6.7) | 14.5 (7.1) | 12.4 (16.2) | 12.7 (14.4) | 12.7 (16.7) | 12.4 (22.3) | |
| DSF | 6.4 (1.4) | 6.9 (1.5) | 6.7 (3.4) | 6.6 (3.1) | 6.8 (3.5) | 6.5 (4.8) | |
| DSB | 4.5 (1.6) | 5.0 (1.7) | 4.8 (3.8) | 4.8 (3.5) | 4.9 (3.9) | 4.6 (5.4) | |
| SFA | 17.2 (7.4) | 20.7 (8.0) | 19.1 (17.9) | 18.7 (16.1) | 18.7 (18.2) | 19.1 (25.1) | |
| SFV | 11.9 (5.3) | 13.5 (5.7) | 12.6 (12.7) | 12.8 (11.4) | 13.3 (12.9) | 12.2 (17.7) | |
| TMTA | 44.8 (28.4) | 38.6 (30.3) | 41.5 (67.6) | 41.9 (60.4) | 39.0 (69.5) | 44.5 (93.7) | |
| TMTB | 125.0 (78.8) | 81.0 (85.1) | 103.1 (188.0) | 102.9 (167.8) | 98.6 (189.9) | 107.4 (261.4) | |

DSB: Digit Span Backward, DSF: Digit Span Forward, LMD: Logical Memory – Delayed recall, LMI: Logical Memory – Immediate recall, SFA: Semantic Fluency – Animals, SFV: Semantic Fluency – Vegetables, TMTA: Trail Making Test – part A, TMTB: Trail Making Test – part B

**Table S7.** Results of Fisher’s exact test analyses for differences in behavioral profiles between MSR and SSR groups stratified by diagnosis (CU vs CI). Values are frequencies of neuropsychiatric symptoms (percentage).

| **NPS** | **SSR (*n* = 36)** | **MSR (*n* = 1005)^a^** | ***p*** |
| --- | --- | --- | --- |
| *Cognitively unimpaired* | *n* = 23 | *n* = 535 |  |
| Delusions (Y/N) | 0/23 (0.0%) | 4/531 (0.7%) | 1.000 |
| Hallucinations (Y/N) | 0/23 (0.0%) | 1/534 (0.2%) | 1.000 |
| Agitation (Y/N) | 0/23 (0.0%) | 20/515 (3.7%) | 1.000 |
| Depression (Y/N) | 1/22 (4.3%) | 49/486 (9.2%) | 0.711 |
| Anxiety (Y/N) | 3/20 (13.0%) | 44/491 (8.2%) | 0.431 |
| Euphoria (Y/N) | 0/23 (0.0%) | 3/532 (0.6%) | 1.000 |
| Apathy (Y/N) | 0/23 (0.0%) | 12/523 (2.2%) | 1.000 |
| Disinhibition (Y/N) | 0/23 (0.0%) | 6/529 (1.1%) | 1.000 |
| Irritability (Y/N) | 0/23 (0.0%) | 49/486 (9.2%) | 0.249 |
| Motor disturbance (Y/N) | 0/23 (0.0%) | 2/533 (0.4%) | 1.000 |
| Night-time behaviors (Y/N) | 1/22 (4.3%) | 36/498 (6.7%)^b^ | 1.000 |
| Appetite disturbance (Y/N) | 2/21 (8.7%) | 10/524 (1.8%)^b^ | 0.084 |
| *Cognitively impaired* | *n* = 13 | *n* = 470 |  |
| Delusions (Y/N) | 1/12 (7.7%) | 44/426 (9.4%) | 1.000 |
| Hallucinations (Y/N) | 0/12 (0.0%)^c^ | 28/442 (6.0%) | 1.000 |
| Agitation (Y/N) | 4/9 (30.8%) | 127/343 (27.0%) | 0.756 |
| Depression (Y/N) | 2/11 (15.4%) | 148/322 (31.5%) | 0.362 |
| Anxiety (Y/N) | 2/11 (15.4%) | 174/296 (37.0%) | 0.147 |
| Euphoria (Y/N) | 1/12 (7.7%) | 16/454 (3.4%) | 0.376 |
| Apathy (Y/N) | 2/11 (15.4%) | 163/307 (34.7%) | 0.235 |
| Disinhibition (Y/N) | 1/12 (7.7%) | 89/381 (18.9%) | 0.478 |
| Irritability (Y/N) | 5/8 (38.5%) | 180/290 (38.3%) | 1.000 |
| Motor disturbance (Y/N) | 1/12 (7.7%) | 64/406 (13.6%) | 1.000 |
| Night-time behaviors (Y/N) | 4/9 (30.8%) | 133/337 (28.3%) | 0.765 |
| Appetite disturbance (Y/N) | 1/12 (7.7%) | 104/366 (21.8%) | 0.315 |

NPS: Neuropsychiatric symptom

^a^ Only 1005 out of 1037 MSR participants had NPI-Q data available

^b^ *n* = 534

^c^ *n* = 12


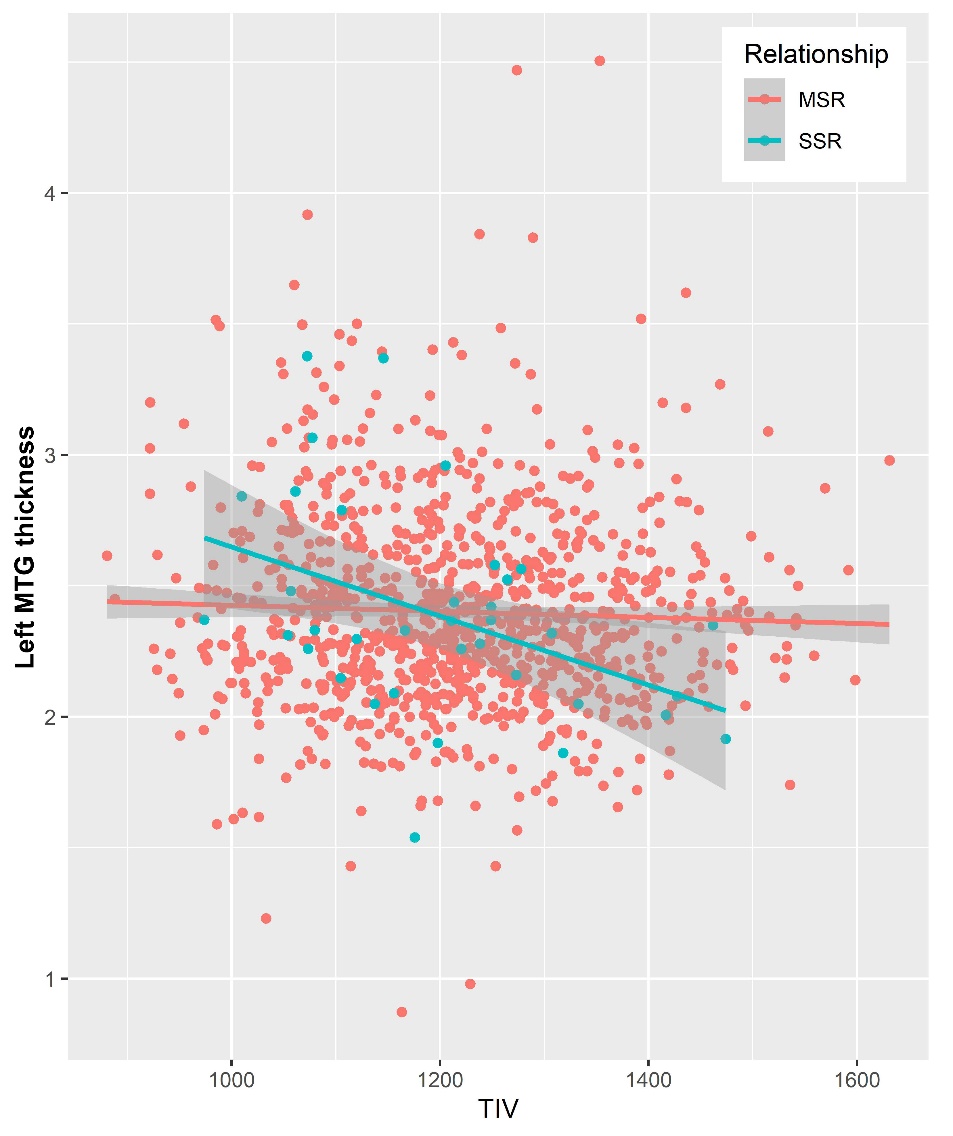


**Figure S1** Differential associations between TIV and the cortical thickness of the left middle temporal gyrus (MTG) in MSR and SSR groups.


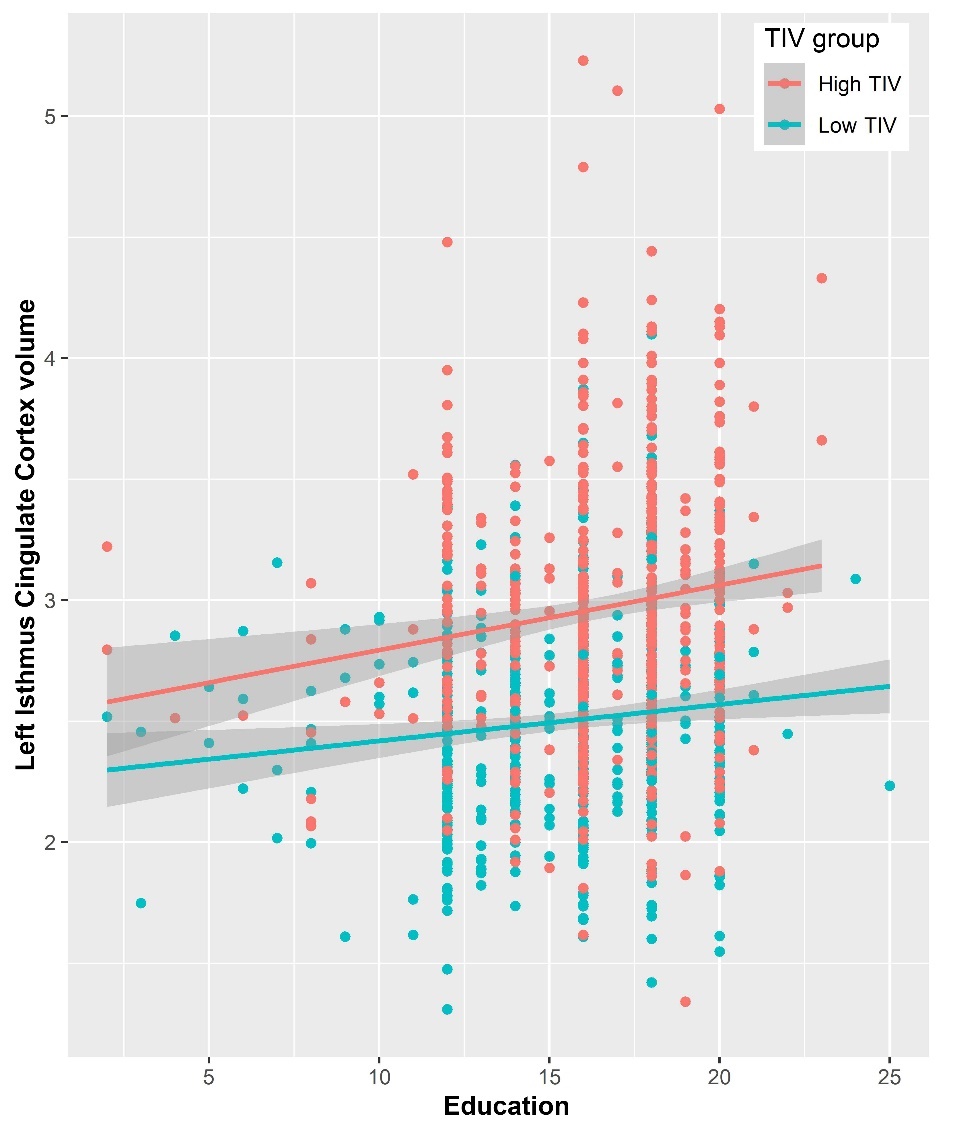


**Figure S2** Differential associations between education and the volume of the left isthmus cingulate cortex in high TIV (ρ = 0.15, *p* < 0.001) and low TIV groups (ρ = 0.11, *p* = 0.007). TIV groups defined by median = 1211.16 ml.


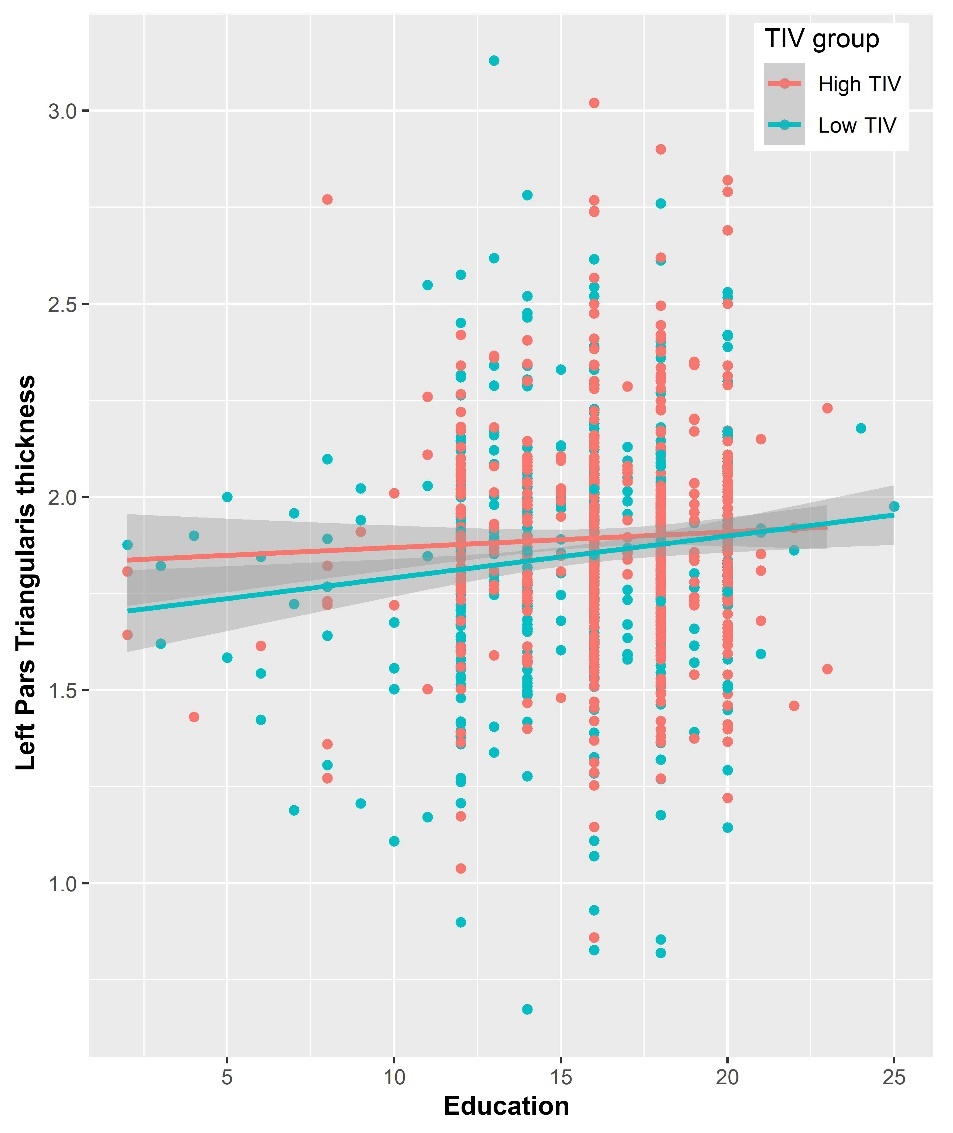


**Figure S3** Differential associations between education and cortical thickness of the left pars triangularis of the inferior frontal gyrus in high TIV (ρ = 0.01, *p* = 0.831) and low TIV groups (ρ = 0.12, *p* = 0.006). TIV groups defined by median = 1211.16 ml.
